# Obstructive sleep apnea is related to alterations in fecal microbiome and impaired intestinal barrier function

**DOI:** 10.1038/s41598-023-27784-0

**Published:** 2023-01-15

**Authors:** Qianjun Li, Ting Xu, Chuan Shao, Wenhui Gao, Mingming Wang, Yongquan Dong, Xiumin Wang, Feijie Lu, Danqing Li, Huanyu Tan, Yin Jiang, Qinge Xie, Fengbo Cai, Lijie Feng, Taoping Li

**Affiliations:** 1 Department of Respiratory and Critical Medicine, Ningbo Yinzhou No. 2 Hospital, Ningbo, 315192 Zhejiang Province China; 2 Sleep Medicine Center, Nanfang Hospital, Southern Medical University, Guangzhou, 510600 China; 3 Department of Pulmonary and Critical Care Medicine, Ningbo Medical Center Lihuili Hospital, Ningbo, China; 4 Department of Special Procurement Ward, The First Affiliated Hospital of Soochow University, Suzhou, China; 5 Department of Cardiovascular, Hangzhouwan Hospital, Ningbo, Zhejiang Province China; 6 School of Pharmacy, Hangzhou Medical College, Hangzhou, China; 7 Department of High-Throughput, Guangzhou Huayin Health Medical Group Co., Ltd, Guangzhou, China

**Keywords:** Microbiology, Physiology, Diseases, Medical research

## Abstract

Obstructive Sleep Apnea (OSA) is related to repeated upper airway collapse, intermittent hypoxia, and intestinal barrier dysfunction. The resulting damage to the intestinal barrier may affect or be affected by the intestinal microbiota. A prospective case–control was used, including 48 subjects from Sleep Medicine Center of Nanfang Hospital. Sleep apnea was diagnosed by overnight polysomnography. Fecal samples and blood samples were collected from subjects to detect fecal microbiome composition (by 16S rDNA gene amplification and sequencing) and intestinal barrier biomarkers—intestinal fatty acid-binding protein (I-FABP) and D-lactic acid (D-LA) (by ELISA and colorimetry, respectively). Plasma D-LA and I-FABP were significantly elevated in patients with OSA. The severity of OSA was related to differences in the structure and composition of the fecal microbiome. Enriched Fusobacterium, Megamonas, Lachnospiraceae_UCG_006, and reduced Anaerostipes was found in patients with severe OSA. Enriched Ruminococcus_2, Lachnoclostridium, Lachnospiraceae_UCG_006, and Alloprevotella was found in patients with high intestinal barrier biomarkers. Lachnoclostridium and Lachnospiraceae_UCG_006 were the common dominant bacteria of OSA and intestinal barrier damage. Fusobacterium and Peptoclostridium was independently associated with apnea–hypopnea index (AHI). The dominant genera of severe OSA were also related to glucose, lipid, neutrophils, monocytes and BMI. Network analysis identified links between the fecal microbiome, intestinal barrier biomarkers, and AHI. The study confirms that changes in the intestinal microbiota are associated with intestinal barrier biomarkers among patients in OSA. These changes may play a pathophysiological role in the systemic inflammation and metabolic comorbidities associated with OSA, leading to multi-organ morbidity of OSA.

## Introduction

Obstructive sleep apnea (OSA) is a common type of sleep breathing disorder, mainly manifested as repeated collapse of the upper airway and cessation of airflow during sleep, leading to intermittent hypoxia (IH) and sleep fragmentation (SF)^1,2^. OSA afflicts nearly 1 billion people worldwide, with the most significant number in China, followed by the United States and Brazil^3^. It is a complex condition with evidence of end-organ morbidity and multi-system dysfunction^4^. However, the pathophysiological mechanism of multiple organ diseases caused by OSA remains unclear. Hereditary and environmental factors are closely related to the inherent phenotypic variation of OSA^5^. However, they account for only a tiny fraction of the phenotypic differences in OSA. The fecal microbiome is the largest genome in the human body. Its disorders are associated with multi-system diseases such as asthma, allergies, obesity, diabetes, inflammatory bowel disease, cardiovascular disease, and increased all-cause mortality^6–8^. The fecal microbiome may also play an essential role in OSA and OSA-induced multi-organ diseases. Alteration in the gut microbiota has been reported in animal models simulating OSA. In the rat model, intestinal microflora imbalance was involved in the occurrence of OSA- induced hypertension^9^. Xue et al. investigated the potential role of OSA-induced intestinal microbiota alterations in atherosclerosis development^10^. However, few studies have directly investigated the gut microbiota in patients with OSA. Only one adult clinical study found that patients with OSA had intestinal flora disturbance^11^. The newer study proposed that some neurological symptoms may be related to gut dysbiosis^12^. Also, changes in the intestinal microbiota can cause circadian clock disturbances, leading to metabolic disorders^13^. Therefore, changes in the intestinal microbiota, in turn, will mediate changes in the host phenotype that manifest as terminal organ disease.

At present, more studies have found that hypoxia is associated with the existence of certain links, such as in the presence of ischemia, hypoxia-induced intestinal dysfunction, the intestinal mucosa epithelium often necrosis, detachment^14,15^. In particular, the disorder is caused by the influence of low oxygen environment factors, and the intensity of free radical reaction increases with the altitude of the plateau, once the intestinal mucosa is damaged, it will make the intestinal flora planted on it inevitably disorder^16^. Lavie found that oxidative stress upregulated HIF-1α and decreased the expression of epithelial tight junction protein in the duodenum, altering the permeability of the intestinal barrier, Hypoxia can induce inflammation, and tissues with inflammatory responses often exacerbate hypoxia, a positive feedback phenomenon^17^. It was found that as the duration of intermittent hypoxia increased, the intestinal mucosa was severely damaged and permeability increased, and this study has reported that intestinal flora displacement can exacerbate structural damage to mesenteric lymph nodes, which in turn increases the level of oxidative stress^18^.

Alterations in intestinal microbiota accompanied by the destruction of the intestinal barrier are another important factor leading to the escalation of systemic inflammation, metabolic disorders^19,20^, and multiple organ failure^21^. The intestinal tract is a microbial-host interface to restrict the escape of intestinal bacteria and toxic media from the intestinal tract. D-lactic acid (D-LA) is the product of the fermentation of gastrointestinal bacteria and served as an indicator of bacterial translocation^22^, intestinal injury, and intestinal permeability^23^. Intestinal fatty acid-binding protein (I-FABP) is a recognized blood marker for early epithelial damage. After an attack of acute intestinal ischemia, inflammation, and hypoxia, this level raised rapidly^19,24^. Plasmatic D-LA and I-FABP are considered to be the most promising and reasonable biomarkers for intestinal barrier impairment^19,25^. Studies of OSA, both in adults^11^ and children^26^, have also shown that these patients have severe intestinal microbiota imbalance. However, evidence from human research linking the microbiome to intestinal barrier dysfunction in OSA is lacking.

Therefore, we look into the relationship between dysbiotic gut microbiome and impaired gut barrier integrity in OSA patients.

## Methods

A prospective case–control study was conducted. Patients aged 18–65 years with suspected OSA were enrolled consecutively in this study. All participants were non-vegetarian, traditional Chinese food. Fecal samples and fasting venous blood were collected the following morning after polysomnography (PSG). No subjects suffered from cardiovascular, cerebrovascular, malignant tumors, respiratory system, hematological system, digestive tract, kidney, or thyroid disease, and no one received continuous positive airway pressure (CPAP) treatment. None of the participants had received antibiotics or microbial modifiers in the two months before enrolment, and none had undergone surgery. The study approval was obtained from the ethics committee of Nanfang Hospital (NFEC No. 2019-091) with the informed consent of each participant.

### Polysomnographic studies

All subjects used the standard PSG for one night, including electroencephalography, electrooculogram, electromyogram, and electrocardiogram. Technologists conducted PSG from 10 p.m. to 7 a.m. Trained Sleep Physician manually scored the PSG data based on the American Academy of Sleep Medicine Guidelines^27^. AHI was summed up times of apnea episodes per hour (airflow decreased ≥ 90% from baseline for at least 10 s) and hypopnea episodes per hour (airflow decreased ≥ 50% for at least 10 s and finger pulse oxygen saturation decreased ≥ 3%). All participants were divided into four groups according to their AHI score: No OSA (AHI < 5 times/hour), mild OSA (5 ≤ AHI < 15 times/hour), moderate OSA (15 ≤ AHI < 30 times/hour), and severe OSA (AHI ≥ 30 times/hour)^28^.

### Sample collection and measurement

After nearly 12 h of fasting (from 8p.m. to 8 a.m. the next day), 1 g of fresh fecal samples were collected in a sterile fecal tube and stored in a refrigerator at -80 °C for subsequent analysis, and fasting venous blood was obtained from 7 a.m. to 8 a.m. after PSG. Two ml of them was collected with a heparin anticoagulant tube, then was centrifuged (3500 × g, 4 °C, 10 min), separated, and stored at − 80 °C for subsequent intestinal barrier function analysis. Other blood indexes such as lipid, glucose were measured by automatic biochemical analyzer (Beckman AU5431, USA). Blood routine was performed using resistance method (Coulter principle) by hematology analyzer (UniCel DxH800, USA).

The inclusion and exclusion criteria were shown as follows:

- Inclusion: Patients aged 18–65 years with suspected OSA; non-vegetarian; traditional Chinese food.
- Exclusion: having suffered from cardiovascular, cerebrovascular, malignant tumors, respiratory system, hematological system, digestive tract, kidney, or thyroid disease; having received continuous positive airway pressure (CPAP) treatment; having received antibiotics or microbial modifiers in the two months before enrolment, having undergone surgery.

I-FABP and D-LA plasma levels were measured by high-sensitivity ELISA (Quantikine; USA; catalog number DFBP20) and Colorimetric Assay (BioVision; USA; catalog number K667) according to product instructions. The detection limits of I-FABP and D-LA were 2.12 pg/mL and 0.01 mM, respectively.

Bead beating isolation was done to extract genomic DNA using the QIAamp DNA Kit (QIAGEN, Hilden, Germany), following the manufacturer's protocol. The extracted DNA concentration was measured by Qubit 2.0 Fluorometer (Life Technology, Carlsbad, CA). DNA quality was detected by 1.0% agarose gel electrophoresis. Then, the 16S rDNA gene regions (V3 and V4) were amplified by PCR. The 16S rDNA libraries were sequenced on the Illumina MiSeq platform PE300 mode (Illumina, USA) using a V3 reagent kit with a 250 bp paired-end run. Sequences were aggregated into operational taxonomic units (OTUs) using 97% identity of the Silva v128 database^29^.

### Bioinformatics and statistical analyses

Quantitative data were displayed by mean ± standard deviation or median (interquartile range) and comparison by Wilcox or Kruskal–Wallis rank-sum test or Mann–Whitney or ANOVA. Qualitative data were displayed by quantity (rates) and comparison by Chi-square test or Fisher’s exact test. Spearman or Pearson correlation tests were utilized to evaluate correlation with continuous variables. Based on 16S data, Alpha(α) diversity index (Observed OTU richness and Shannon Diversity) and Beta(β) diversity index based on Principal Coordinate Analysis (PCoA) (unweighted-Unifrac distance, Adonis) was used to assess the differences between groups. Linear discriminant analysis (LDA) and LDA effect size (LEfSe) (LEfSe 1.0) was used to assess the taxonomic differences among groups. Multiple regression was used to analyze the relationship between clinical variables (such as gender, age, BMI, AHI) and the microbiota characteristics identified in the severe OSA group. The network was then utilized to present the relationship between fecal microbiome, intestinal barrier biomarkers, and AHI (Cytoscape_v3.9.1). Statistical analysis of data was conducted in SPSS (version 21.0) and R software (v3.5.1). P < 0.05 indicates that the difference was statistically significant.

### Ethical approval and consent to participate

The approval was obtained from the ethics committee of Nanfang Hospital (NFEC No.2019-091). The procedures used in this study adhere to the tenets of the Declaration of Helsinki. Informed consent was obtained from each participant in the study.

## Results

**Figure 1:**
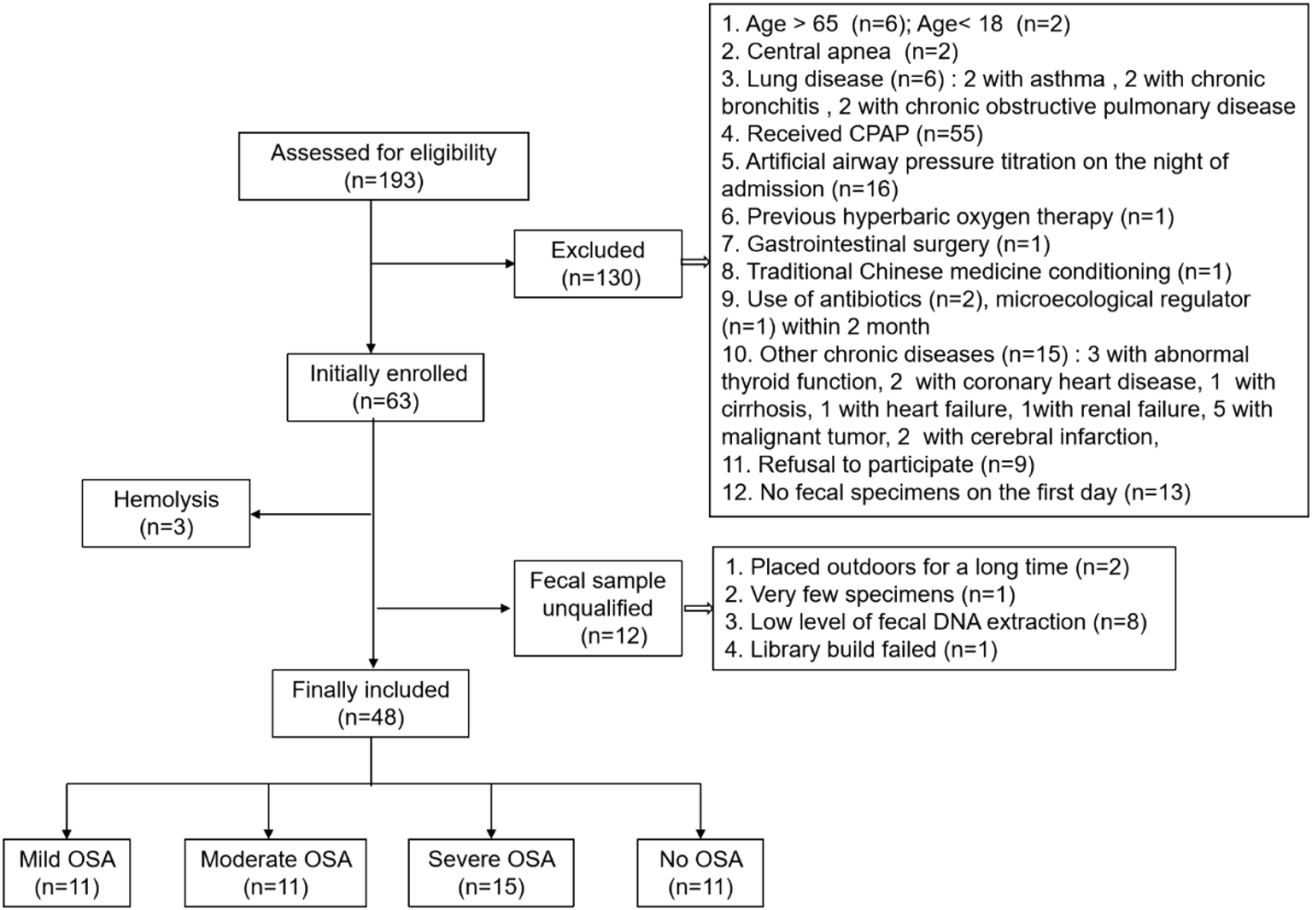
Patient flow chart.

A total of 193 patients were screened from May 2019 to November 2019 in our sleep center, and 48 were eventually included in the analysis (Fig. 1). The prevalence of OSA was high (37/48 patients; 77%) in our sleep center, and 31% of participants were severe OSA. OSA was positively correlated with males, AHI, and BMI, and negatively correlated with SaO2. Levels of intestinal barrier markers were greater among different OSA categories than the control subjects (*P* ≤ 0.01), except for the I-FABP in the mild group. Levels of D-LA were significantly related to I-FABP (Spearman rho = 0.429, *p* = 0.002). But there were no significant differences in age, hypertension, or diabetes (Table 1).

**Table 1:** Baseline characteristics and intestinal barrier markers of participants.

Factor	Non OSA (n = 11)	Mild OSA (n = 11)	Moderate OSA (n = 11)	Severe OSA (n = 15)	P
Demographics					
Age (years)	38(33–40)	38(33–49)	40(35–51)	35(30–45)	0.542
Gender (males)	7(64%)	6(55%)	9(82%)	15(100%)*	0.014
BMI (Kg/m^2^)	24.01 ± 3.11	26.33 ± 2.29	26.49 ± 3.68	28.72 ± 3.38*	0.006
Hypertension (%)	0(0%)	2(18%)	3(27%)	4(27%)	0.302
Diabetes (%)	0(0%)	1(9%)	1(9%)	4(27%)	0.266
PSG date					
AHI (events/h)	2.1 (1.5–2.5)	6.6(5.4–10.3)*	18.7(16.7–20.6)*	59.1 (54.6–73.0)*	< 0.001
Mean SaO_2_ (%)	95 (95–97)	95(94–96)	95(94–96)	93(88–94)*	< 0.001
Lowest SaO_2_ (%)	90(86–93)	87(83–88)	82(75–85)*	63(52–75)*	< 0.001
Intestinal barrier markers					
D-LA (mmol/L)	1.40(0.01–2.74)	7.73(4.19–11.52)*	7.88(6.09–10.58)*	8.74(5.94–16.36)*	< 0.001
I-FABP (pg/mL)	1112.55 ± 346.91	1443.33 ± 530.17	1914.30 ± 845.35*	2055.32 ± 880.91*	0.010

BMI, body mass index; AHI, apnea–hypopnea index; SaO_2_, pulse oxygen saturation; D-LA, D-lactic acid; I-FABP, intestinal fatty acid-binding protein.

values are expressed as mean ± standard deviation, median (interquartile range) or number (percentage).

p values based on Kruskal–Wallis or Chi-Squared (Fisher's Exact Test) or ANOVA test.

*p values significant for the comparison of OSA groups versus No OSA Subjects.

### Microbiota characteristics in groups

**Figure 2:**
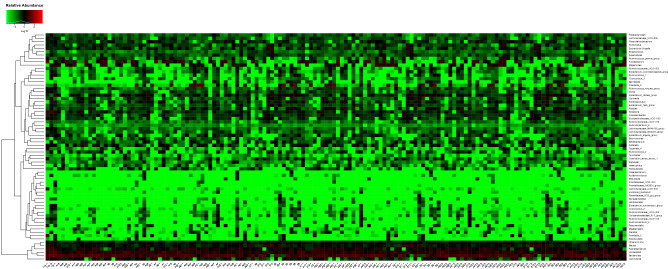
Heatmap of 48 stool samples at the genus level. Stool samples demonstrate the high relative abundance of Bacteroides, Faecalibacterium, Blautia, Blautia, and Lachnospira(relative abundance ≥ 0.5% were annotated, < 0.5% were classified as others).

A total of 3980179 paired-end reads were attained, with an average of 117491 nonchimera tags and an average of 421 OTUs per sample. Heat map based on the fecal samples of most abundant taxa at the genus level showed gut microbiota had a high relative abundance of Bacteroides, Faecalibacterium, Blautia, and Lachnospira in most samples (Fig. 2).

**Figure 3:**
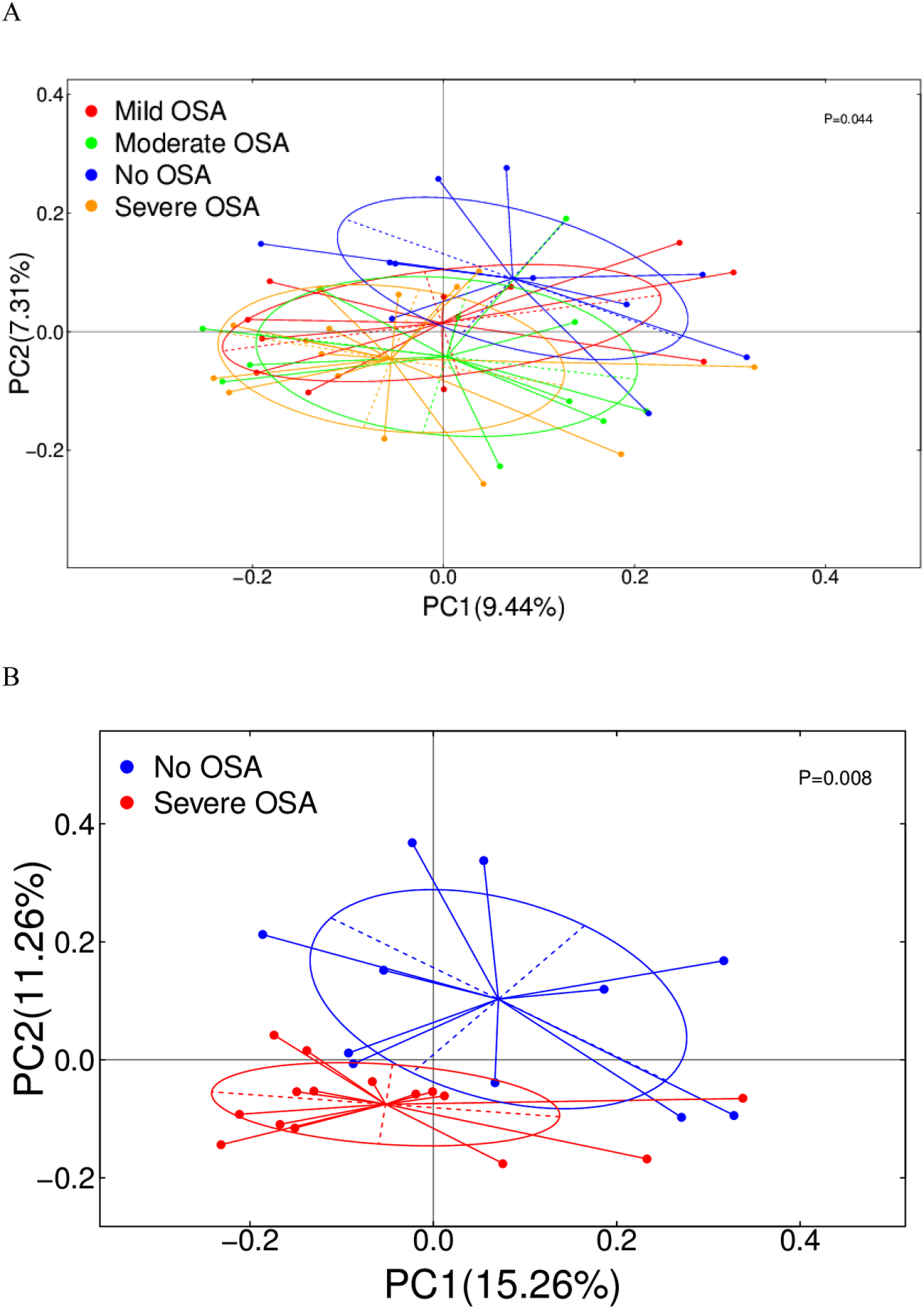
β diversity differences in different severity groups of OSA and severe OSA. PCOA (Unweigther-Unifrac distance) between (**A**) different severity groups of OSA versus no OSA (Adonis P = 0.044) and (**B**) severe OSA versus no OSA (adonis P = 0.005).

Then, we assessed differences in the fecal microbiome between the participants with various degrees of OSA severity compared with no OSA. α diversity analysis showed no differences in Observed OTU richness and Shannon diversity index between control subjects and OSA (Supplementary Figure S1). Other species diversity indices (Simpson) and richness (ACE, Chao) were also not statistically significant (Supplementary Table S1). β diversity analysis based on PCoA showed significant differences in the composition of OSA with varying severity (Fig. 3A; Adonis *p* = 0.044). Further pairwise analysis (OSA group with different severity compared with no OSA group) showed that the microbiome characteristics of severe OSA subjects were significantly separated from those of no OSA (Fig. 3B; Adonis *p* = 0.008). Simultaneously, differences in the other OSA and no OSA groups were similar (Supplementary Figure S2). Unweighted uniFrac distance was an indicator of the degree of compositional similarity between samples. The closer the samples were, the more similar the samples' species composition was, and vice versa.

### Taxa differences between groups

**Figure 4:**
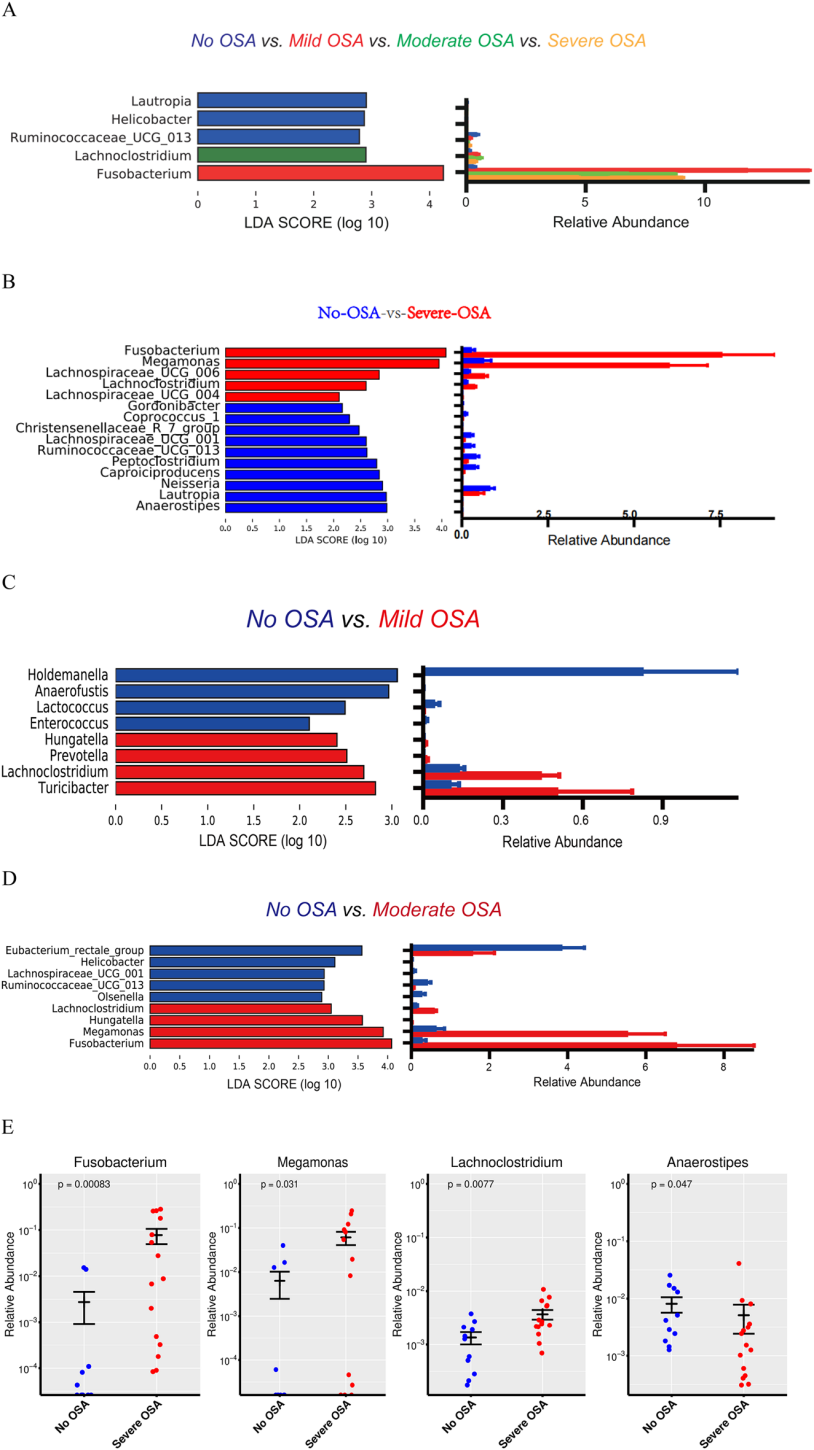
Taxa differences in different severity OSA. LEfSe identifies microbiota differences in (**A**) Different severity groups of OSA versus no OSA (Kruskal–Wallis), (**B**) severe OSA versus no OSA (Wilcox). (**C**) Mild OSA versus no OSA(Wilcox). (**D**) moderate OSA versus no OSA (Wilcox). (**E**) Scatter plot of the bacteria at the genus level with relative abundance greater than 0.5% in all samples in severe OSA participants (Wilcox).

To identify taxa differences of the gut bacteria, LDA and LEfSe were used. The gut microbiota on the top four genera from different severity of OSA subjects was enriched with Fusobacterium, Lachnoclostridium and decreased with Ruminococcaceae_UCG_013 when compared to no OSA subjects (Fig. 4A, Supplementary Figure S3A). The microbiota of patients with severe OSA was enriched with Fusobacterium, Megamonas, Lachnospiraceae_UCG_006 and decreased with Anaerostipes (Fig. 4B, Supplementary Figure S3B). The mild group was enriched with Turicibacter and Lachnoclostridium, decreased with Holdemanelle and Enterococcus (Fig. 4C, Supplementary Figure S3C). While in moderate OSA was increased with Fusobacterium, Megamonas, and Lachnoclostridium, decreased with Eubacterium_rectale_group (Fig. 4D, Supplementary Figure S3D). The top four genera's taxa with relative abundance greater than 0.5% in all samples among severe OSA were further analyzed by a Scatter plot of relative abundance (Fig. 4E). At the phylum level, Fusobacteria showed significant differences among the four groups (*P* = 0.0173). However, Firmicutes, Bacteroides, Actinobacteria, and Proteobacteria had no significant differences (Supplementary Table S2, nor did the Firmicutes (F) to Bacteroides(B) ratio (Supplementary Figure S4).

### Correlation and regression analyses

To estimate the potential relationship between clinical variables and the microbiota characteristics with relative abundance greater than 0.5% in all samples of severe OSA, we conducted Spearman analysis. Significant associations between microbiota characteristics and AHI, SaO_2_, BMI, glucose, TG, TCHO, HDL, LDL and gut epithelial barrier markers were found. The D-LA level was positively correlated with the abundance of Lachnoclostridium, Megamonas and Fusobacterium, and negatively correlated with Peptoclostridium and Anaerostipes (Table 2). While I-FABP level was negatively correlated with Peptoclostridium. In addition, Peptoclostridium was significantly correlated with Neutrophils and Monocytes (Table 2). To further explore the independent relationship between severe OSA and the microbiota characteristics mentioned above, we conducted multiple linear regression analysis. Significant association with severe OSA was identified. Higher AHI predicted higher relative abundance of Fusobacterium (B = 0.538, P = 0.041) and Peptoclostridium (B = − 0.491, P = 0.046), independent of age, BMI, and sex (Table 3).

**Table 2:** Correlation analysis of variables and microbiota characteristics of severe OSA.

	Anaerostipes		Peptoclostridium		Lachnoclostridium		Megamonas		Fusobacterium	
	r	p	r	p	r	p	r	p	r	p
AHI (events/h)	− 0.484	0.012*	− 0.601	0.001*	0.368	0.064	0. 616	0.001*	0.606	0.001*
Mean SaO_2_ (%)	0.445	0.023*	0.510	0.008*	− 0.426	0.030*	− 0.363	0.068	− 0.524	0.006*
Lowest SaO_2_ (%)	0.480	0.013*	0.564	0.003*	− 0.445	0.023*	− 0.485	0.012*	− 0.596	0.001*
Glucose (mmol/L)	− 0.402	0.042*	− 0.104	0.611	0.312	0.120	0.162	0.429	0.093	0.651
TG (mmol/L)	− 0.123	0.548	− 0.426	0.030*	0.370	0.063	0.146	0.476	0.346	0.083
TCHO (mmol/L)	0.065	0.754	0.125	0.544	− 0.099	0.631	− 0.456	0.019*	− 0.088	0.668
HDL (mmol/L)	0.128	0.543	0.484	0.014*	− 0.435	0.030*	− 0.255	0.219	− 0.333	0.104
LDL (mmol/L)	0.065	0.757	0.089	0.671	− 0.106	0.615	− 0.443	0.027*	− 0.044	0.834
Leukocytes (10^9^/L)	0.048	0.815	− 0.378	0.057	0.085	0.679	0.219	0.282	0.150	0.465
Neutrophils (10^9^/L)	0.067	0.746	− 0.477	0.014*	0.123	0.550	0.128	0.534	0.253	0.211
Monocytes (10^9^/L)	− 0.206	0.313	− 0.470	0.015*	0.214	0.295	0.344	0.085	0.281	0.164
Lymphocytes (10^9^/L)	0.266	0.190	− 0.199	0.329	0.162	0.429	0.351	0.079	0.136	0.507
D-LA (mmol/L)	− 0.409	0.038*	− 0.491	0.011*	0.500	0.009*	0.408	0.039*	0.522	0.006*
I-FABP (pg/mL)	− 0.304	0.131	− 0.467	0.016*	0.266	0.189	0.247	0.223	0.241	0.236
BMI (Kg/m^2^)	− 0.313	0.120	− 0.644	< 0.001*	0.423	0.031*	0.545	0.020*	0.285	0.158

AHI, apnea hypoventilation index; M-SaO_2_, Mean pulse oxygen saturation; L-SaO_2_, Lowest pulse oxygen saturation; Glucose, fasting blood sugar; TG, triglycerides; TCHO, total cholesterol; HDL, high density lipoprotein; LDL, low-density lipoprotein; Leukocytes, white blood cell count; Neutrophils, neutrophil count; Monocytes, mononuclear cell count; Lymphocytes, lymphocyte count; D-LA, D-lactic acid; I-FABP, intestinal fatty acid-binding protein; BMI, body mass index.

*p* values based on Spearman analysis.

*P < 0.05, the difference is statistically significant.

**Table 3:** Multiple regression analysis between microbiota signatures of severe OSA and baseline characteristics.

	Anaerostipes		Peptoclostridium		Megamonas		Fusobacterium	
	B	*P*	B	*P*	B	*P*	B	*P*
AHI	− 0.402	0.146	− 0.491	0.046	0.358	0.156	0.538	0.041
BMI	0.263	0.341	− 0.133	0.577	0.271	0.285	− 0.312	0.226
Sex	− 0.088	0.724	− 0.131	0.550	0.106	0.646	− 0.135	0.562
Age	− 0.175	0.405	0.224	0.227	− 0.016	0.935	− 0.134	0.492

BMI, body mass index; AHI, apnea–hypopnea index.

### Intestinal barrier biomarkers and microbiota

**Figure 5:**
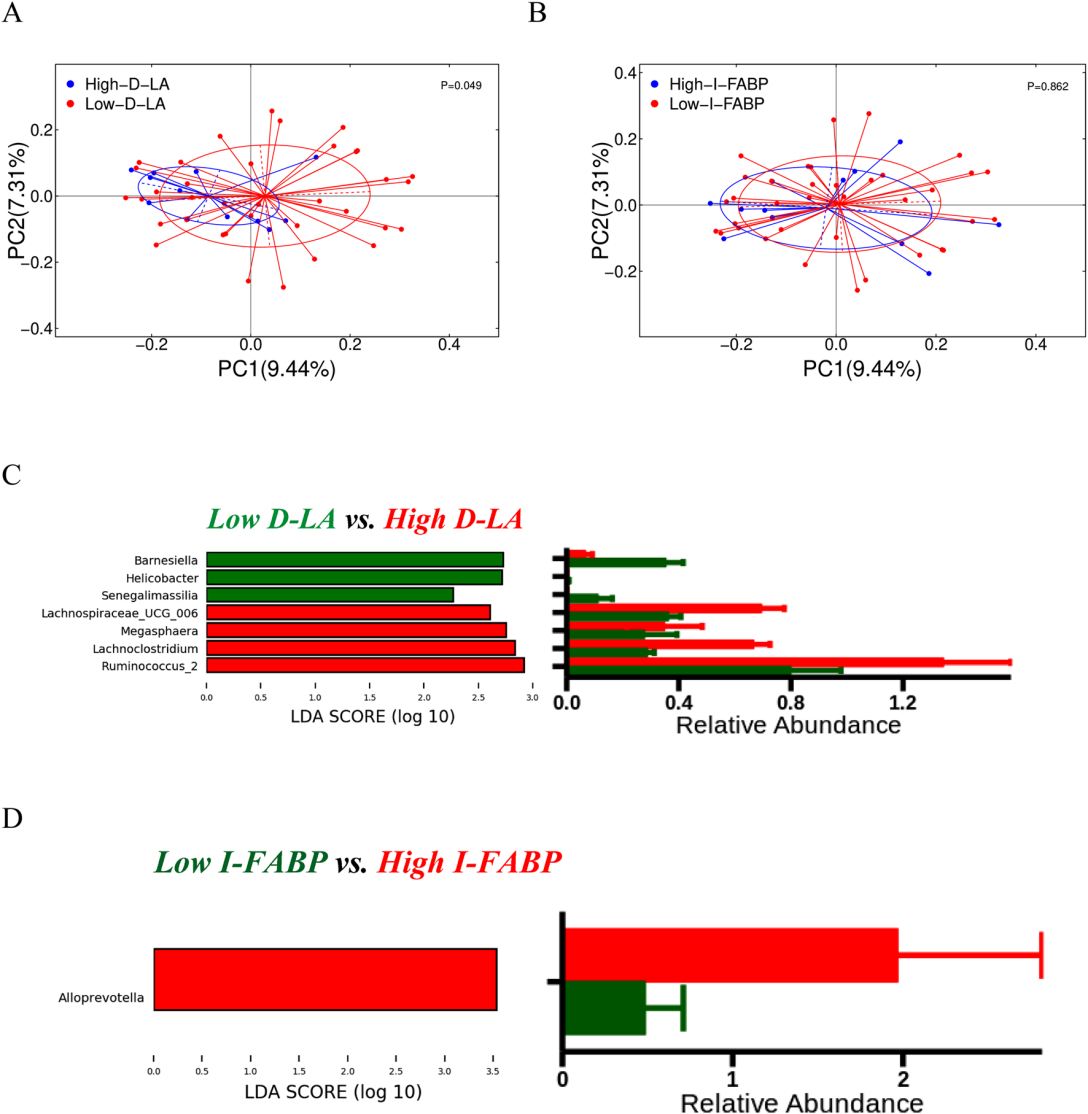
Correlation between changes in intestinal microbiota and intestinal barrier dysfunction. Beta diversity between (**A**) high versus low D-LA (Adonis p = 0.049) and (**B**) high versus low I-FABP levels (Adonis p = ns). (**C**) The gut microbiota with high D-LA was enriched with Ruminococcus_2, Lachnoclostridium, and Lachnospiraceae_UCG_006 and decreased with Senegalimascilia (Wilcox). (**D**) The gut microbiota with high I-FABP was enriched with Alloprevotella (Wilcox).

To investigate the possible relationship between intestinal barrier dysfunction and intestinal microbiota dysfunction, we evaluated the association of changes in gut microbes with high and low values of intestinal barrier biomarkers. High and low D-LA and I-FABP subgroups were defined. The high biomarker subgroup has the biomarker value in the fourth quartile (highest 25%) and low biomarker subgroup has the value in the third quartile (0–75%). PCoA based on Unweighted-Unifrac had a significant difference in the composition of intestinal flora with high and low D-LA (Fig. 5A; Adonis *P* = 0.049), while there was no difference in α diversity (Supplementary Figure S5). There was no difference in both α and β diversity among I-FABP groups (Supplementary Figure S5B, Fig. 5B). LEfSe analysis showed differentially enriched taxa between groups. Samples with high D-LA were enriched with Ruminococcus_2, Lachnoclostridium, and Lachnospiraceae_UCG_006 and decreased in Senegalimascilia (Fig. 5C). Samples with high I-FABP were enriched with Alloprevotelle (Fig. 5D). Therefore, this study demonstrated a relationship between the enrichment of intestinal microbiota and the intestinal barrier in OSA.

### Network analysis

**Figure 6:**
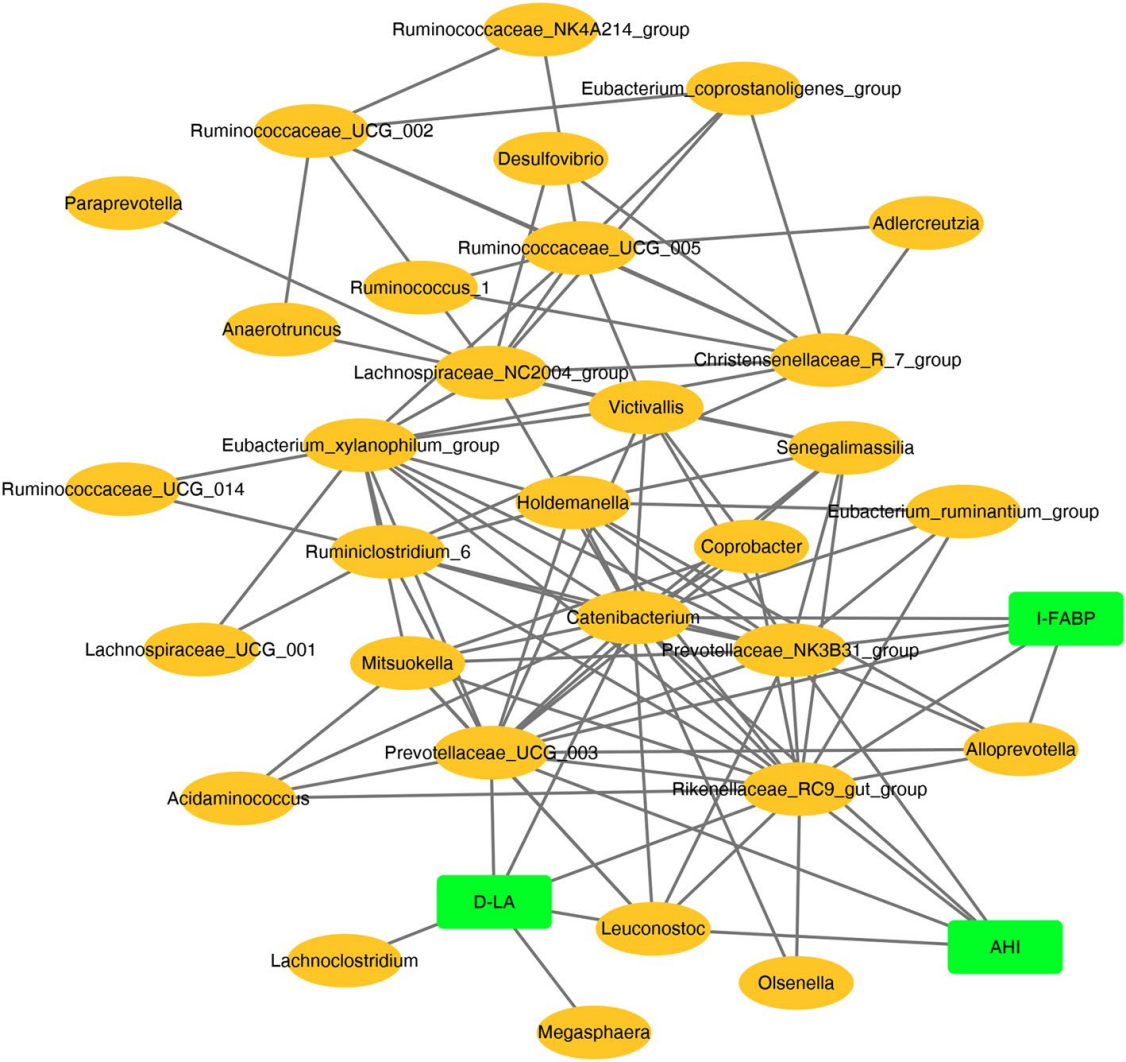
Network between taxa, AHI, and intestinal barrier biomarkers. SparCC was used to construct a genus taxa network. Positively related variables were kept in the network (The correlation coefficient between bacteria and clinical indicators was ≥ 0.4, and between bacteria and bacteria was ≥ 0.7). AHI, D-.LA, and I-FABP were associated with unique taxa. Streptobacillus, Prevotellaceae_UCG_003, and Rikenellaceae_RC9_gut_group were the common genera. Gut commensals co-occurred AHI and intestinal barrier biomarkers (D-LA and I-FABP).

To further explore the relationship of gut microbiota, intestinal barrier, and AHI, we built a co-occurrence network at the genus level (> relative abundance 0.5% species). The graph represented features that were positively correlated. Network analysis identified the taxa of defined meta-communities that tend to coexist in the gut microbiota. One metacommunity was characterized by co-occurrence between Ruminococaceae_NK4A214_group, Olsenella, Ruminococcaceae_UCG_014, and Alloprevotella. AHI, D-LA, and I-FABP were associated with unique intestinal flora, respectively. Streptobacillus, Prevotellaceae_UCG_003, and Rikenellaceae_RC9_gut_group were the mutual genera of the three (Fig. 6). Thus, supporting that intestinal microbiota was related to OSA and intestinal barrier.

In addition, the co-occurrence network between intestinal barrier, AHI and microbiome in different severity levels (mild/moderate/severe OSA) was shown in Supplementary Fig. S6. The chart shows that in mild OSA, Megamonas, Peptoclostridium and Senegalimassilia are the three reciprocal genera (Supplementary Figure S6A); in moderate OSA, Alloprevotella is the three reciprocal genera (Supplementary Figure S6B). In the severe OSA, Lachnospiraceae_UCG_001, Ruminiclostridium_6 and Eubacterium_xylanophilum_group were three intergeneric genera (Supplementary Figure S6C). The severity co-occur with the different species genera, which deserves further studies to follow.

## Discussion

OSA is a systemic, comprehensive disease with cardiovascular disease and metabolic abnormalities. IH plays an important role in OSA and its associated multi-organ pathology, but its mechanism of action is not fully understood. This study confirmed that changes in the gut microbiome were associated with gut barrier biomarkers in patients with OSA, and that the dominant genera of severe OSA were also associated with glucose, lipids, neutrophils, monocytes and BMI. Network analysis identified associations between the gut microbiome, gut barrier biomarkers, and AHI. These changes may play a pathophysiological role in the systemic inflammation and metabolic comorbidities associated with OSA, leading to the multi-organ morbidity of OSA.

The present investigation found an association between OSA and increased intestinal barrier biomarkers that are also associated with unique microbiome profiles, with D-LA levels positively correlated with Lachnoclostridium, Megamonas, and Fusobacterium and negatively correlated with Peptoclostridium and Anaerostipes. I-FABP levels were negatively correlated with Peptoclostridium. β-Diversity showed significant differences in microbial community composition between high and low D-LA groups, with high plasma D-LA positively correlated with Ruminococcus_2, Lachnoclostridium, Lachnospiraceae_UCG_006 positively and Senegalimascilia negatively, whereas fecal microbiota high in I-FABP were enriched in Alloprevotella. This suggests that impairment of the epithelial barrier is associated with enrichment in specific microbiota. Increased intestinal barrier alterations in OSA have been well described, such as circulating blood I-FABP, D-LA and lipopolysaccharide-binding protein are increased^23,30,31^. We previously reported that circulating D-LA and I-FABP are significantly elevated in OSA compared with healthy subjects^19^.

Based on our present study and reports in the relevant literature, we speculate that repeated hypoxia may be a cause of epithelial damage that can lead to dysbiosis of the intestinal flora, and that these adverse factors may impair intestinal function by increasing intestinal permeability. Repeated hypoxia is responsible for epithelial damage that can lead to gut dysbiosis. Recurrent hypoxia and reoxygenation could impair intestinal function by increasing intestinal permeability, bacterial translocation, and decreasing the integrity of tight junctions^25,26^. Increased gut barrier alterations in OSA have been well described, such as increased I-FABP, D-LA, and Lipopolysaccharide-Binding Protein in circulation blood^18,27,28^. A mouse model of sleep apnea had shown that intestinal colonization by Prevotella and Desulfovibrio contributed to increased intestinal permeability^29^. In humans, D-lactate producing bacteria, such as Streptococcus and Enterococcus, have been found in faces of patients with chronic fatigue syndrome^12^. Furthermore, the significant increase in intestinal bacterial translocation products (endotoxin and D-lactic acid) was associated with systemic inflammation and could predict adverse cardiovascular events^30^. We previously reported that circulating D-LA and I-FABP were significantly elevated in OSA compared to healthy subjects^14^. This survey also found a relationship between OSA and an increase in gut barrier biomarkers. These biomarkers were also related to unique microbiome profiles. D-LA level was positively correlated with Lachnoclostridium, Megamonas and Fusobacterium, and negatively correlated with Peptoclostridium and Anaerostipes. The level of I-FABP was negatively correlated with Peptoclostridium. The β-diversity showed a significant difference in microbial community composition between high and low D-LA groups. High plasma D-LA was positively correlated with Ruminococcus_2, Lachnoclostridium, Lachnospiraceae_UCG_006, and negative with Senegalimascilia, while the microbiota of fecal with high I-FABP was enriched with Alloprevotella. It indicated that the damaged epithelial barrier was related to the enrichment of specific microbiota. Fusobacterium and Lachnoclostridium are both differential bacteria, which were confirmed again in both DESeq and LEFse methods.

In addition, hypoxia has a direct effect on intestinal gut dysbiosis. Morenos-Indias et al. found significant changes in intestinal microbial community structure in an animal model mimicking OSA^32^. IH exposed in mice models cumulatively disrupted the fecal microbiome and metabolism^33^. These animal models, analogous to those of chronic OSA, showed an alteration in the gut microbiota compared to normal level of oxygen. In humans, an increased intestinal oxygen level also affects the composition of fecal and mucosal adherent microbiota (e.g., Proteobacteria and Actinobacteria)^30^. Repeated hypoxia due to upper airway obstruction can also have local manifestations in all blood perfused organs^34^. Therefore, microbiome changes may occur in different mucous membranes in OSA. For example, alterations in the nasal microbiome are related to OSA and inflammatory biomarkers^35^. Lung microbiota in OSA is distinct from that of control subjects^36^. Oral microbiota is significantly disturbed in pediatric patients with OSA, leading to OSA-related metabolomics^37^. IH and SF, two essential characteristics of OSA, also established a hypoxic environment in many parts of the digestive tract^38^. Albenberg et al. observed that oxygenation of the host influences gut luminal oxygenation, altered the composition of the gut microbiology^30^. Reduced oxygen in the intestine enabled obligate anaerobic microorganisms(such as Proteobacteria and Actinobacteria) to become more competitive and overgrowth^30^. Simultaneously, increased tissue oxygenation can directly affect microorganisms, such as reducing anaerobes^39^. Therefore, the gut may provide a unique environment conducive to living aerobic and facultative anaerobic organisms.

In our study, OSA was related to alterations in β-diversity of the fecal microbiome. Among OSA subjects with different degrees of severity, the intestinal flora of Fusobacterium and Lachnoclostridium were enriched, while Ruminococcaceae_UCG_013 was reduced compared to no OSA. In severe OSA, Fusobacterium, Megamonas, and Lachnospiraceae_UCG_006 were abundant, while Ruminococcaceae_UCG_013 was reduced. It was consistent with Ko C. et al., the relative abundance of the Ruminococcaceae was greater in the control group^11^. While the relative abundances in Megamonas were contrary to Ko C. et al. This may be due to different DNA extraction kits, different PCR amplification primers and clustering methods, or the participants ' living areas and lifestyles are different, or our samples are relatively small. However, not only did we find an enrichment of Megamonas in the severe group, but also an increase in the moderate group. In contrast, Ko C. et al. only found a decrease in Megamonas in the severe group. Future clinical studies are needed to validate our results. Then, Fusobacterium and Peptoclostridium demonstrated an independent relationship with severe OSA, which may be useful in identifying subjects at risk of gut microbiome disorders related to OSA. Some of the above fecal microbiome differences, such as Lachnoclostridium and Lachnospiraceae_UCG_006, were also associated with intestinal barrier biomarkers. Moreover, the differences in microbiota signatures of the moderate group were very close to those of the severe group. Thus, it supported the reasonableness of comparing the moderate and severe OSA groups as a single OSA group with the no OSA group, without including the mild group in the study^40^.

In addition, microbiota characteristics in severe OSA was not only significantly correlated with sleep parameters, but also associated with lipids and BMI. Anaerostipes was correlated with fasting blood glucose and Peptoclostridium was associated with neutrophils and monocytes. Therefore, alterations of these microbiota could cause low-grade chronic inflammation^41^, immune and metabolic abnormalities. Fusobacterium has been linked to cardiovascular disease^42^, colorectal cancer^43^, oral and lung infections^44,45^. Megamonas is correlated with chronic kidney disease^46^. Alloprevotella has been associated with infection and diabetes^47,48^. Thus, Changes in these microbes may play an important role in multi-system and multi-organ disease triggered by OSA. Our study also found that the microbiota characteristics in the severe OSA group were significantly correlated with the intestinal barrier biomarkers. The co-occurrence network analysis identified an association between the fecal microbiome, intestinal barrier biomarkers, and AHI. These results suggested that intestinal barrier dysfunction was associated with bacterial dysfunction in OSA.

Recently, Mashaqi S, in his review of OSA and gut dysbiosis, found that most studies were conducted in animal models with an increase in the F/ B ratio^41^. Also, among children, the ratio of F/B was higher in snorers than in controls^26^. Unlike previous animal models and children’s studies, we did not find significant differences in α diversity or F/B proportion among patients with OSA. However, our results are consistent with another adult OSA study^11^. This may be because humans and animals have different gut populations. Collado et al. enrolled snoring children through a questionnaire, did not perform PSG test, which lacked the ability to diagnose snoring or OSA in children without this test. Furthermore, children's intestine may not be fully developed^49^, and their dietary structure and tolerance to hypoxia may differ from adults'. More studies on the intestinal flora of adult OSA are needed in the future to support our findings.

The advantages of this study include not only clinical evaluation of the relationship between intestinal microbiota and OSA but also novel evaluation of the association with haemoconcentration of D-LA and I-FABP. We supported the pathophysiological role of intestinal flora and changes in the intestinal barrier in OSA. However, there are some disadvantages. The first disadvantage is that the included sample size is relatively small. Although the intestinal flora in OSA was statistically significantly different from no OSA, no differences in α and β diversity were observed in mild and moderate OSA. Second, we did not collect detailed dietary habits of participants and their physical activity that might affect the gut microbiome^50,51^. Third, we focused on the most important sleep parameters, other sleep indicators such as sleep stages and sleep efficiency were not included, and these needs to be taken into account in the future. In addition, we did not evaluate the effects of microbiota modification strategies (e.g., CPAP, microecological modulators) on intestinal barrier biomarkers and intestinal microbiota. Future study may require more longitudinal, interventional investigations to assess causality. With the important role of precision medicine in the development of individualized disease treatment protocols. The approach of gut microbial composition and metabolic components will also become an important part of the individualized treatment of OSA. The present study provides a scientific basis for the involvement of intestinal flora in OSA and pathogenesis, and how OSA regulates host metabolism through intestinal flora will be worthy of further study, and there are few reports of related clinical studies at home and abroad. For this reason, further studies in multicenter and large sample clinical trials are needed in the future.

## Conclusion

In this study, we used human specimens to construct a model to further demonstrate that OSA can cause significant alterations in the structure of the intestinal flora, thereby changing the homeostatic relationship between host and intestinal flora, and ultimately can even lead to multi-organ damage as well as disease. This finding may provide clinical opportunities to develop targeted therapies for patient-specific microbiota profiles.

## Supplementary Information

Supplementary Information 1.

Supplementary Information 2.

Supplementary Information 3.

Supplementary Information 4.

## Data Availability

The datasets discussed during the current study are deposited in NCBI's Sequence Read Archive (SRA) and are accessible through SRA Series accession number PRJNA851918. Other datasets generated or analysed during this study are included in this published article and its supplementary information files.
