# Supplementary material for: Obstructive sleep apnea is related to alterations in fecal microbiome and impaired intestinal barrier function
Source: Sci Rep. 2023 Jan 15;13:778. doi: 10.1038/s41598-023-27784-0 (PMC9841009; doi:10.1038/s41598-023-27784-0)
Supplement: Supplementary file 1 — Supplementary Information 1. [file 41598_2023_27784_MOESM1_ESM.doc]

**Supplementary Tables**

**Supplementary Table S1. Comparison of other α diversity in four groups**

| Alpha | Non OSA  (n=11) | Mild OSA (n=11) | Moderate OSA (n=11) | Severe OSA (n=15) | P |
| --- | --- | --- | --- | --- | --- |
| Chao | 518.70±222.16 | 550.06±181.07 | 523.44±175.47 | 547.77±175.91 | 0.977 |
| Ace | 517.44±222.14 | 552.95±173.46 | 513.49±168.46 | 545.57±172.82 | 0.933 |
| Simpson | 0.09±0.06 | 0.11±0.06 | 0.11±0.05 | 0.19±0.07 | 0.546 |

Note: The values in the table are expressed as mean ± standard deviation，and Kruskal-Wallis test is used for comparison.

**Supplementary Table S2. Difference in abundance at the phylum level**

| species | Non OSA  (n=11) | Mild OSA (n=11) | Moderate OSA (n=11) | Severe OSA (n=15) | P |
| --- | --- | --- | --- | --- | --- |
| Actincbacteria | 4.44±4.82 | 0.92±0.83 | 2.65±3.73 | 1.27±1.90 | 0.1495 |
| Proteobacteria | 4.54±3.64 | 4.95±3.64 | 5.00±2.90 | 5.47±3.25 | 0.8118 |
| Firmicutes | 51.63±22.07 | 43.89±13.01 | 39.78±12.30 | 42.81±15.17 | 0.5353 |
| Bacteroideres | 38.99±26.95 | 38.02±8.86 | 45.60±16.31 | 42.15±17.64 | 0.7771 |
| Fusobacteria | 0.27±0.61 | 11.85±16.18 | 6.80±11.77 | 8.24±11.16 | 0.0173 |

Note: The values in the table represent mean ± standard deviation, and Kruskal-Wallis test is used.

**Supplementary Figures**

A B


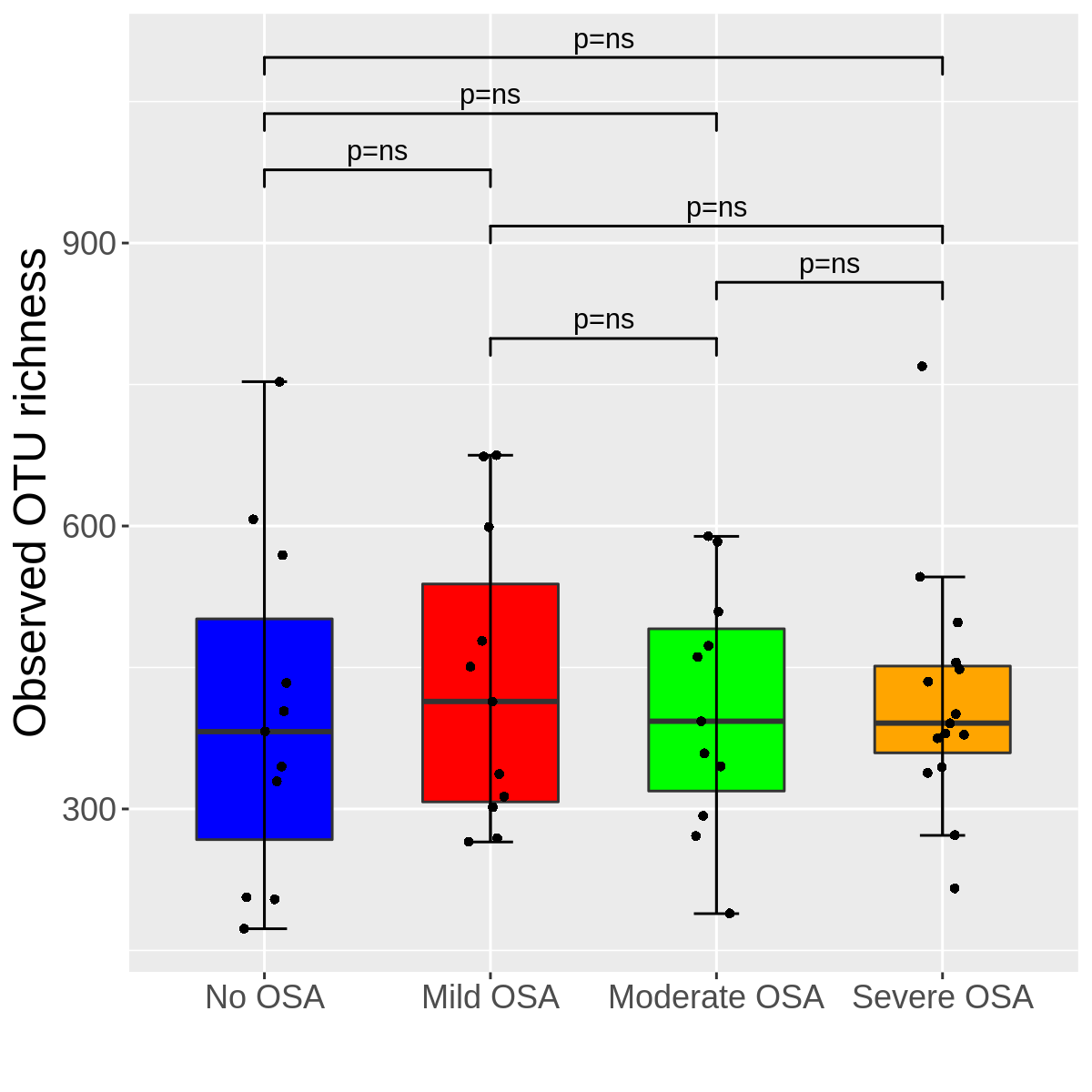

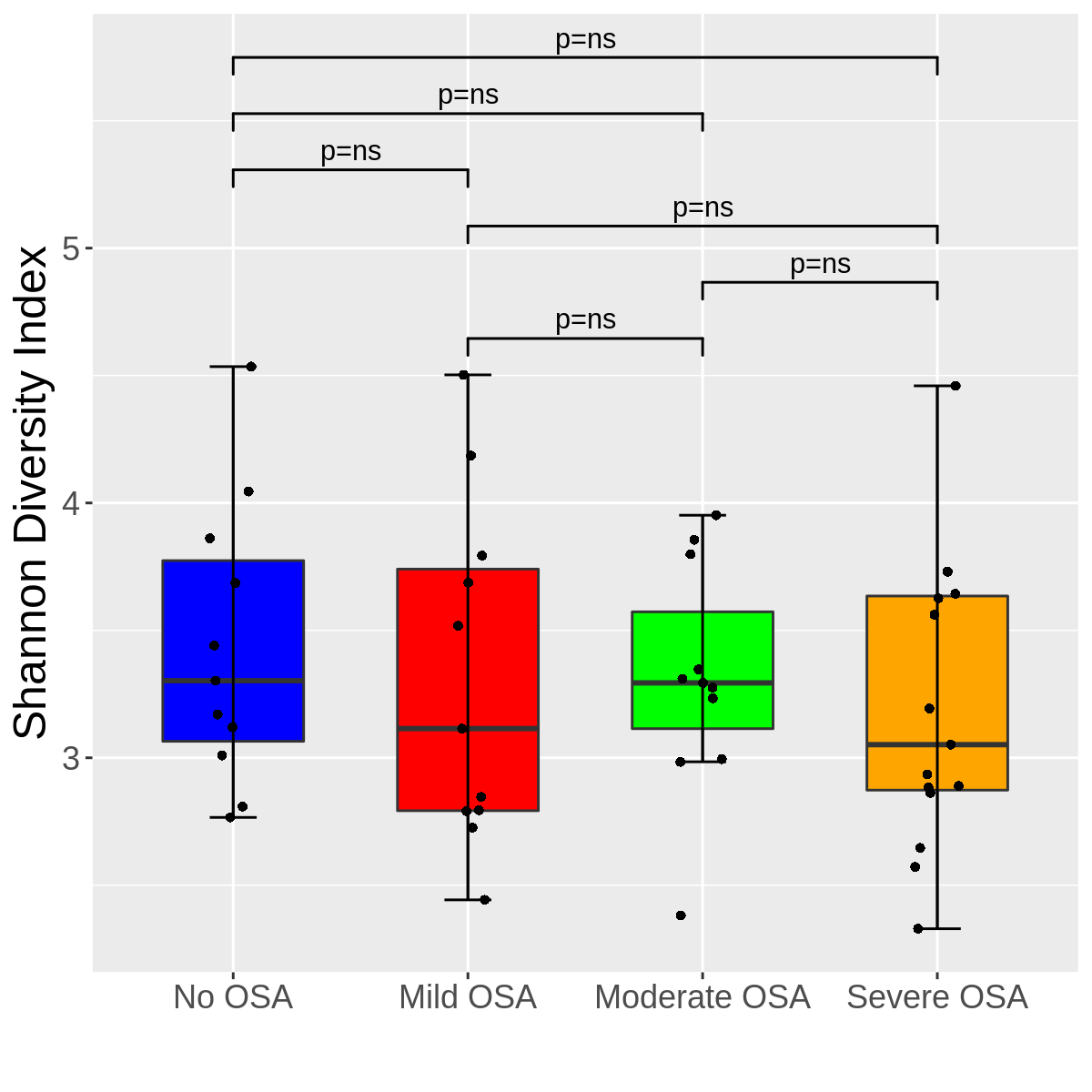


**Supplementary Figure S1.α and β diversity of the participants.** Differences in (A) Observed operational taxonomic units (OTUs) richness (Kruskal-Wallis P=ns) and (B)Shannon Diversity Index (Kruskal-Wallis P=ns) between four groups.

A B


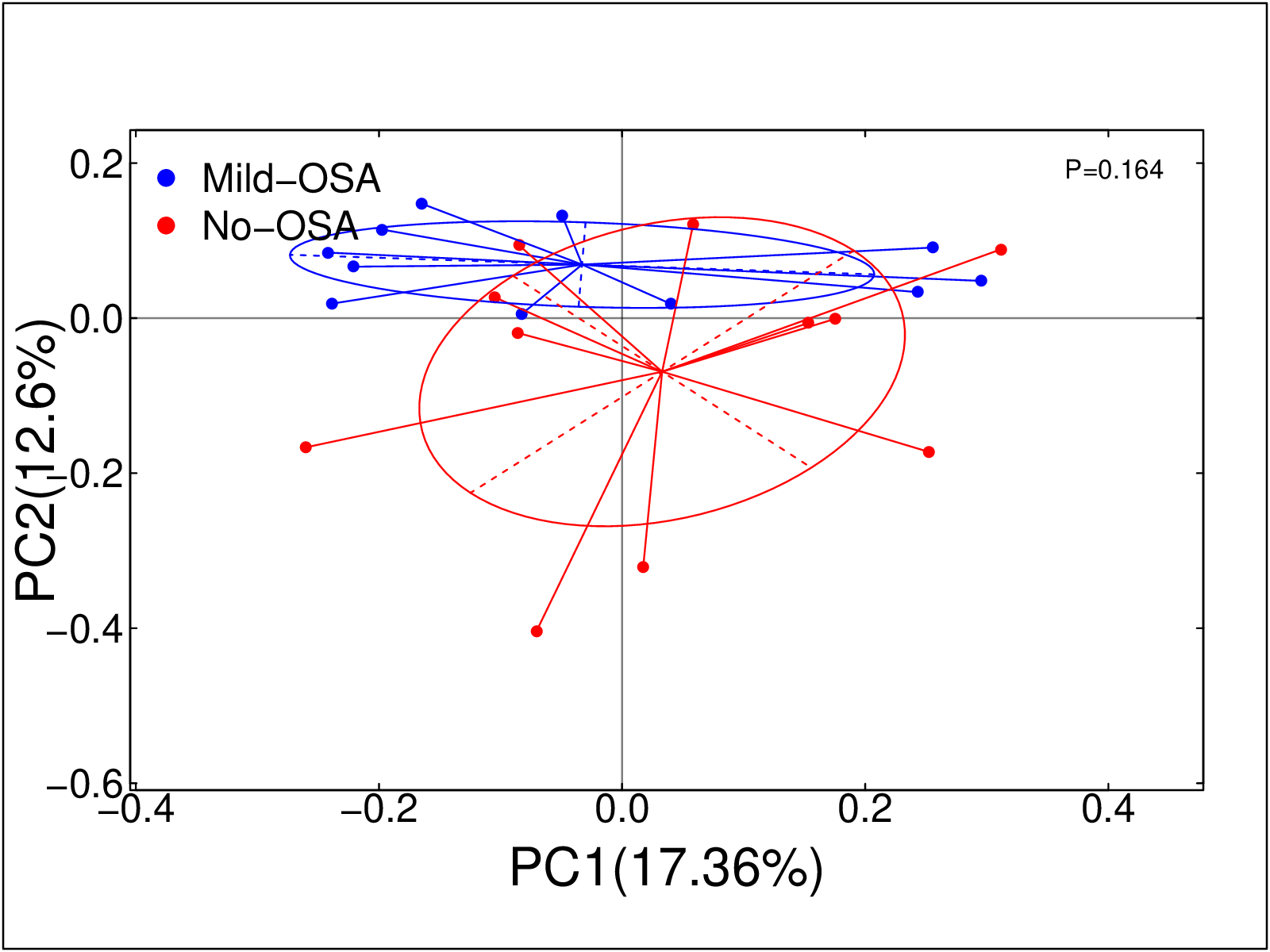

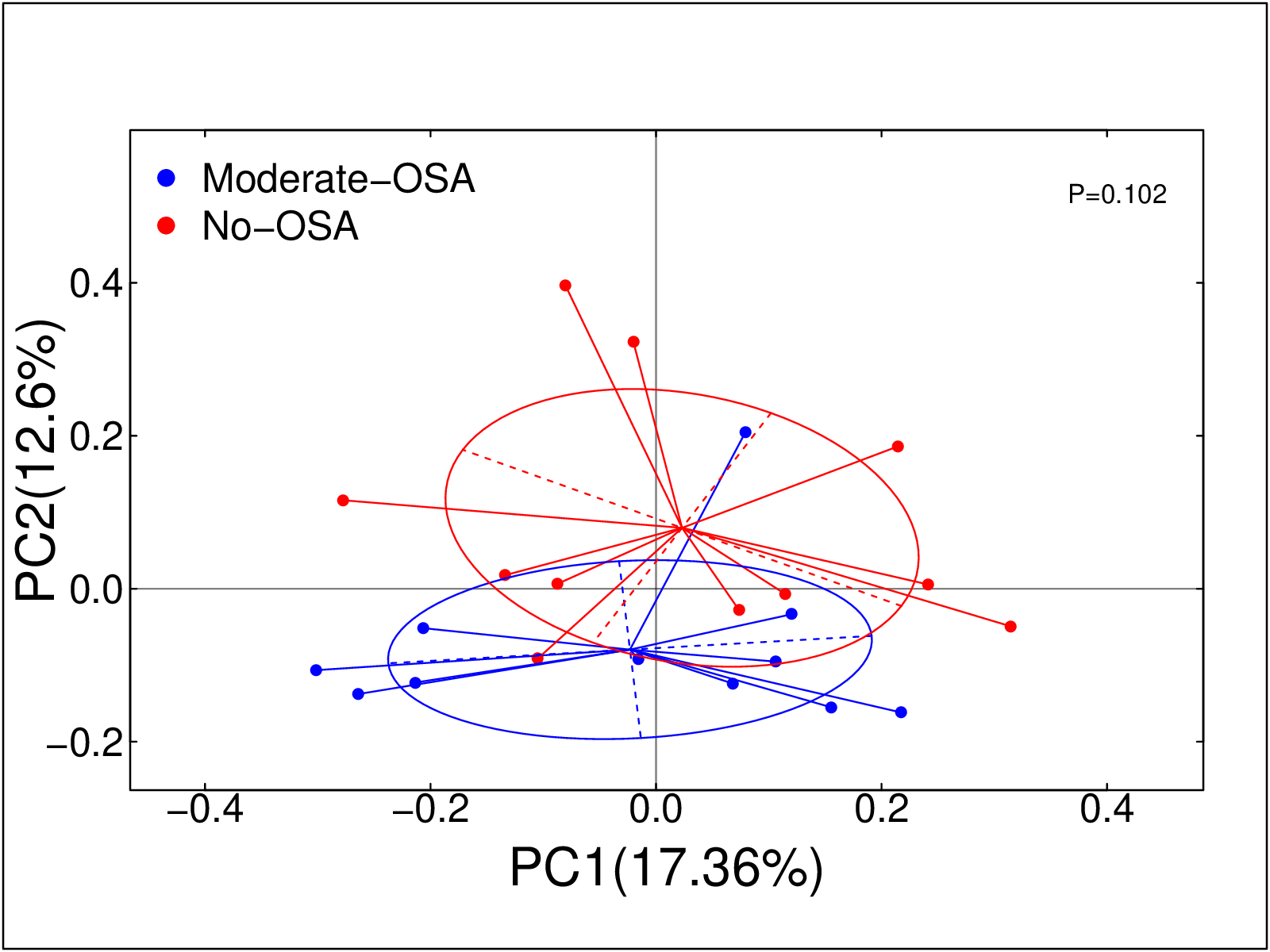


**Supplementary Figure S2. β diversity differences in mild and moderate OSA.** PCOA (Unweigther-Unifrac distances) between (A) Mild OSA vs. No OSA. (B) Moderate vs. No OSA.

**A**


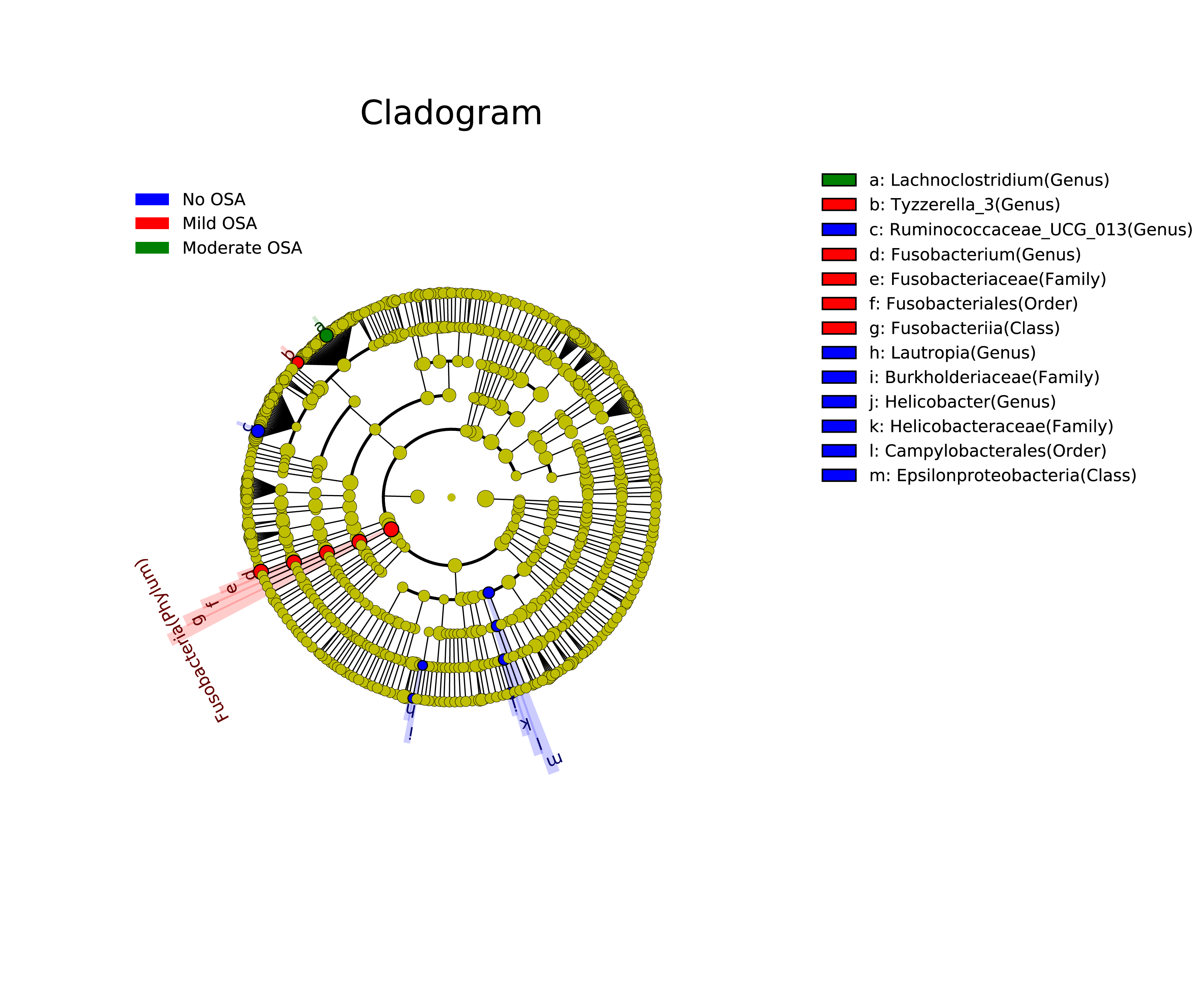


**B**


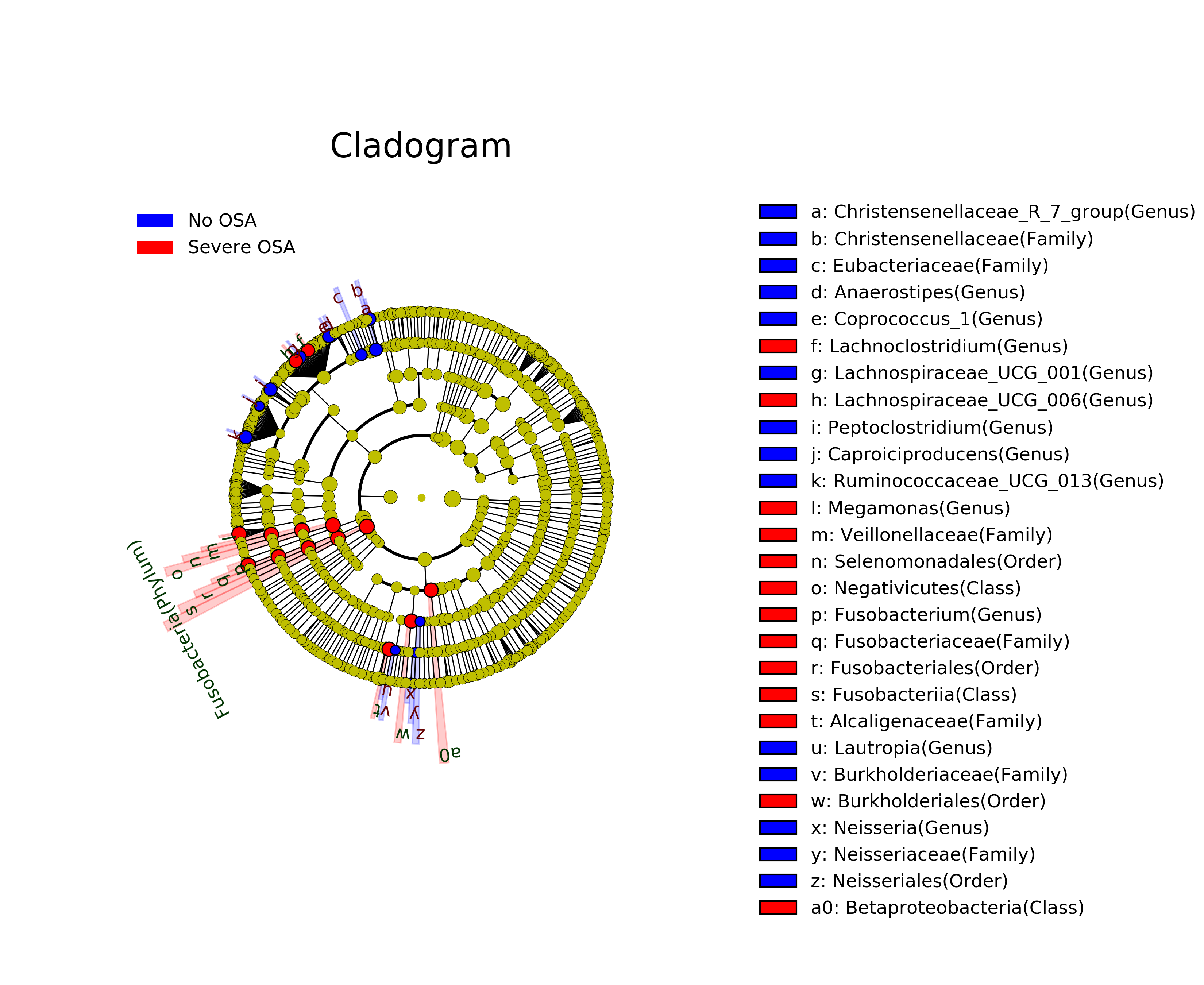


C


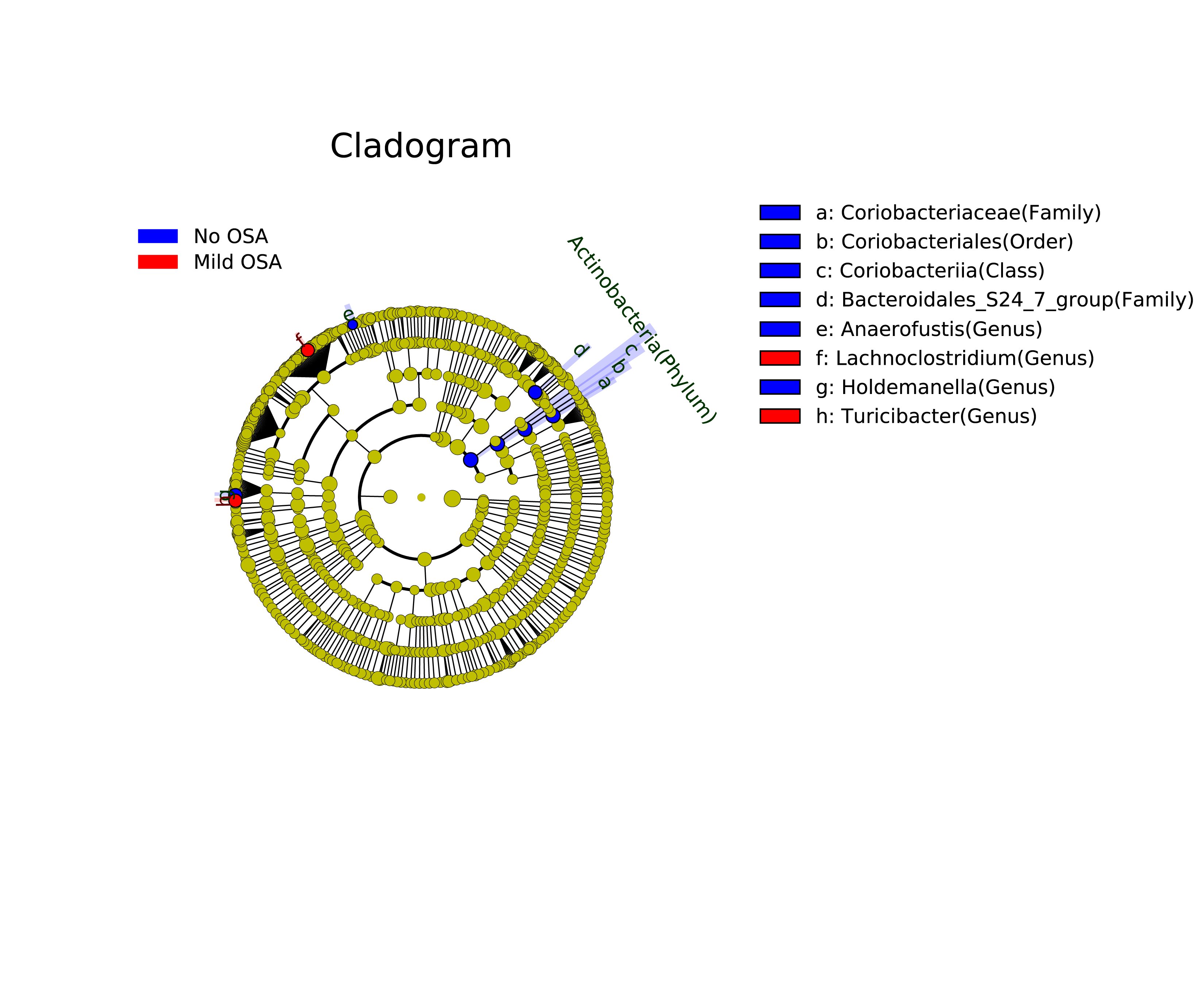


D


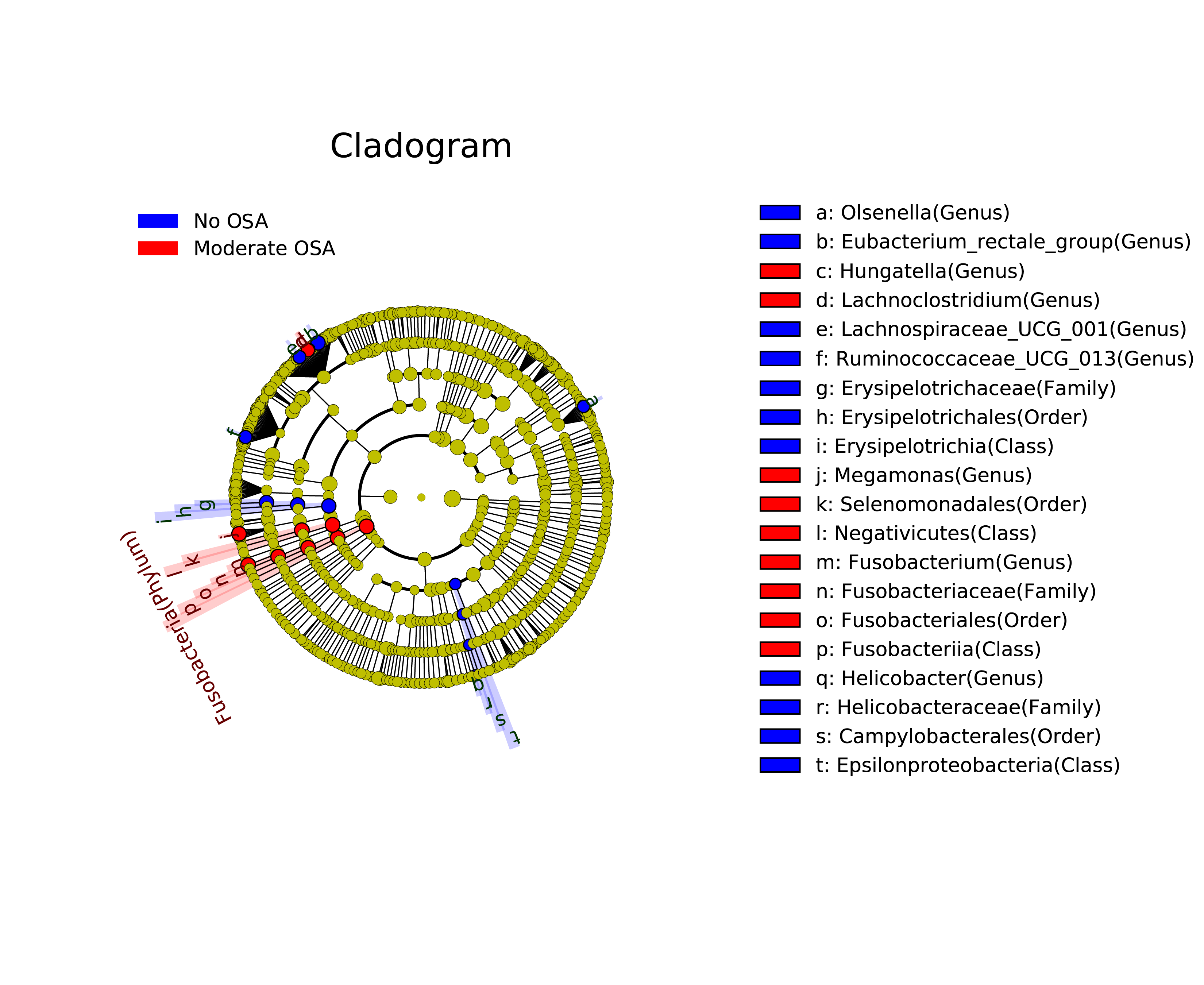


**Supplementary Figure S3.** **Taxa differences in different severity OSA.** Cladogram identify microbiota differences between (A)Different severity groups of OSA vs. no OSA, (B) severe OSA vs. no OSA. (C) mild OSA vs. no OSA. (D) moderate OSA vs. no OSA.


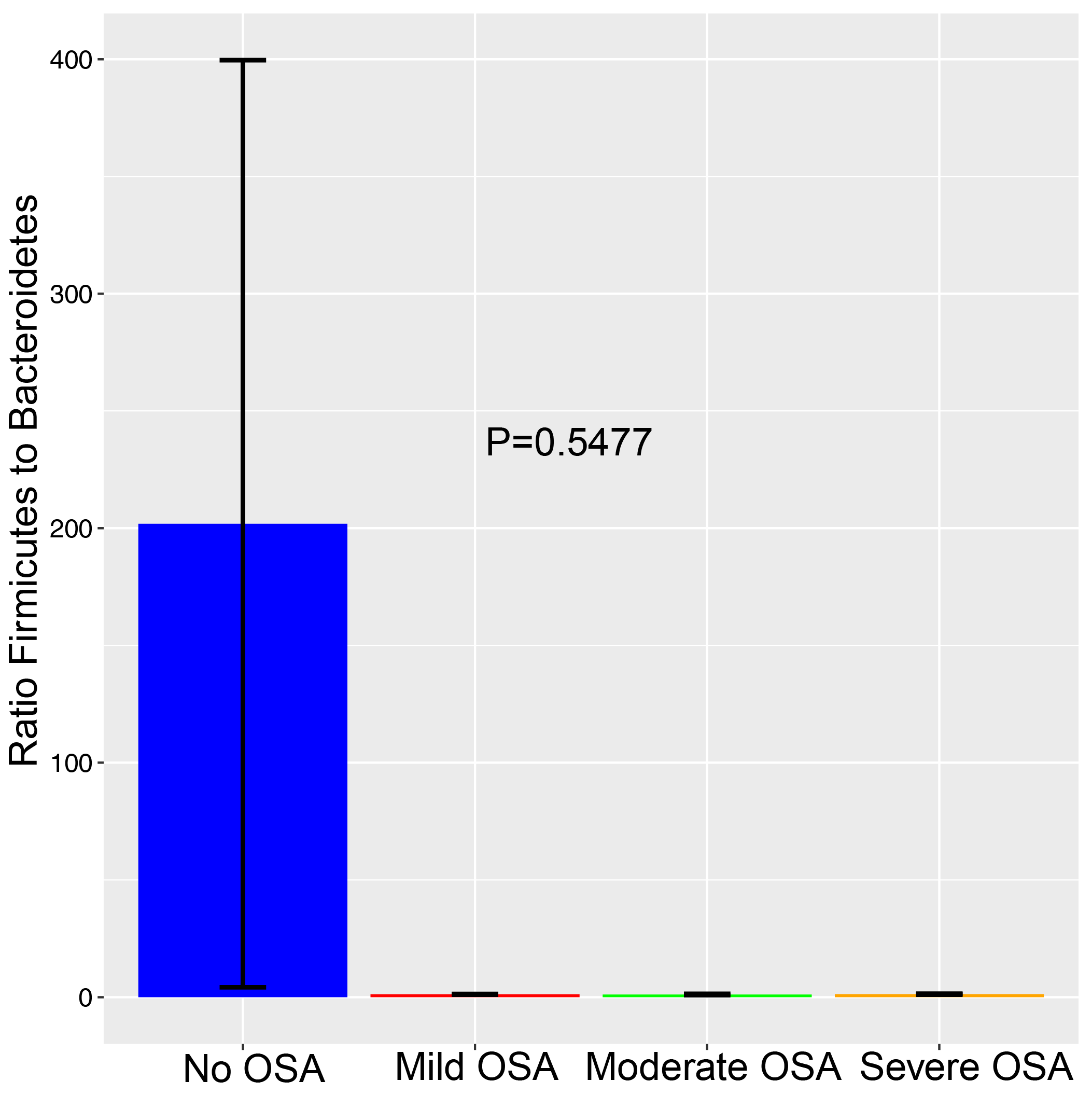


**Supplementary Figure S4. The proportion of Firmicutes to Bacteroidetes at phylum level among four groups**.

A


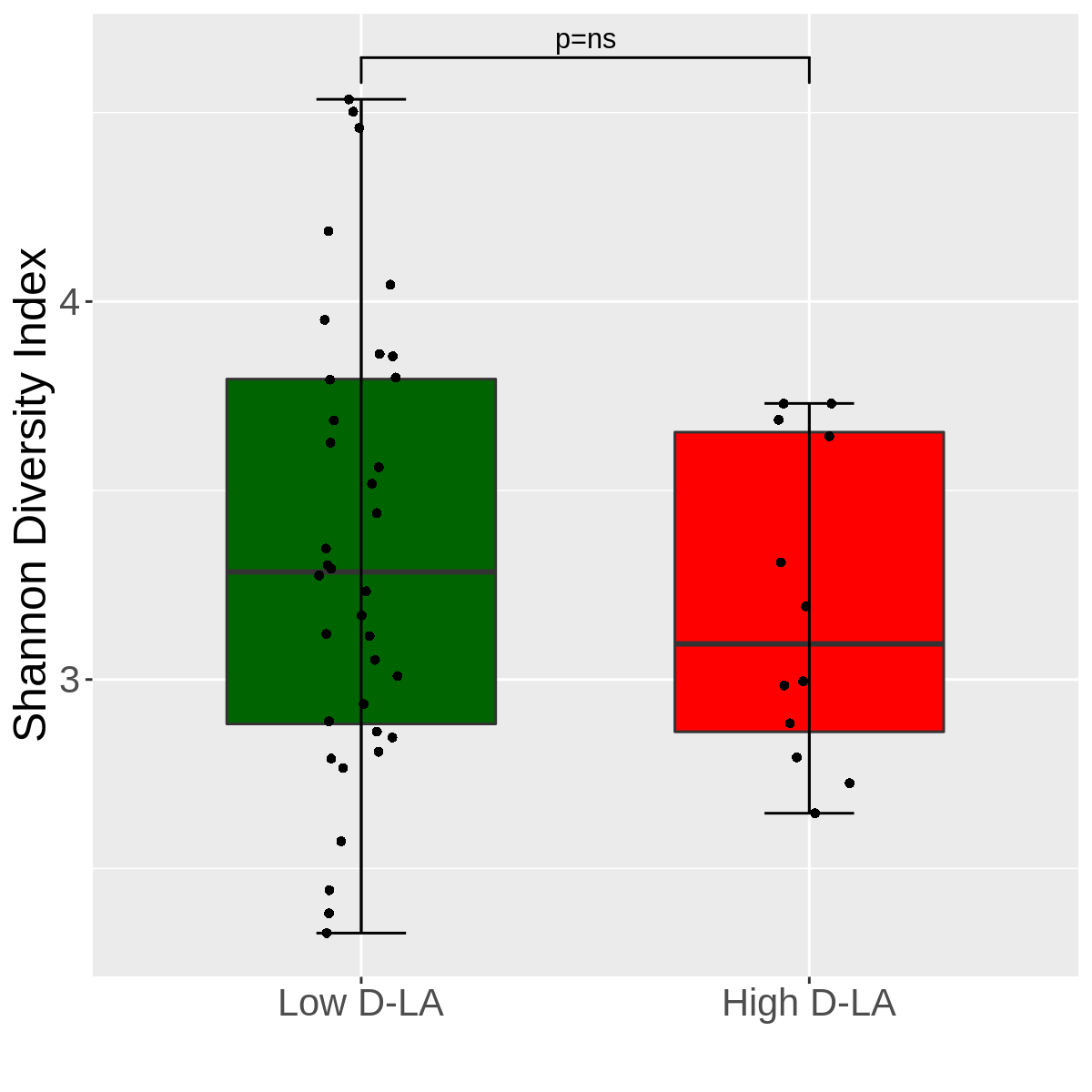

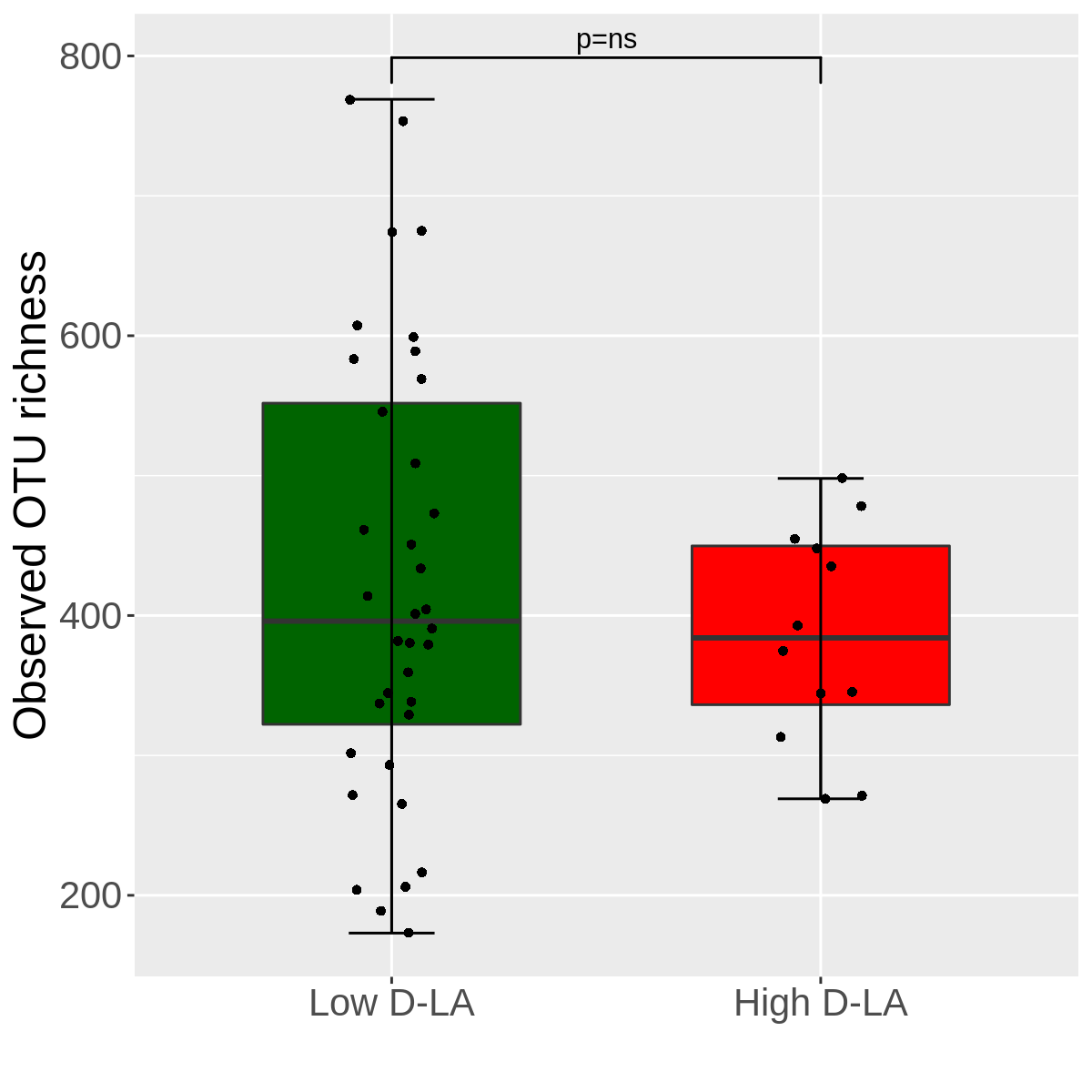


B


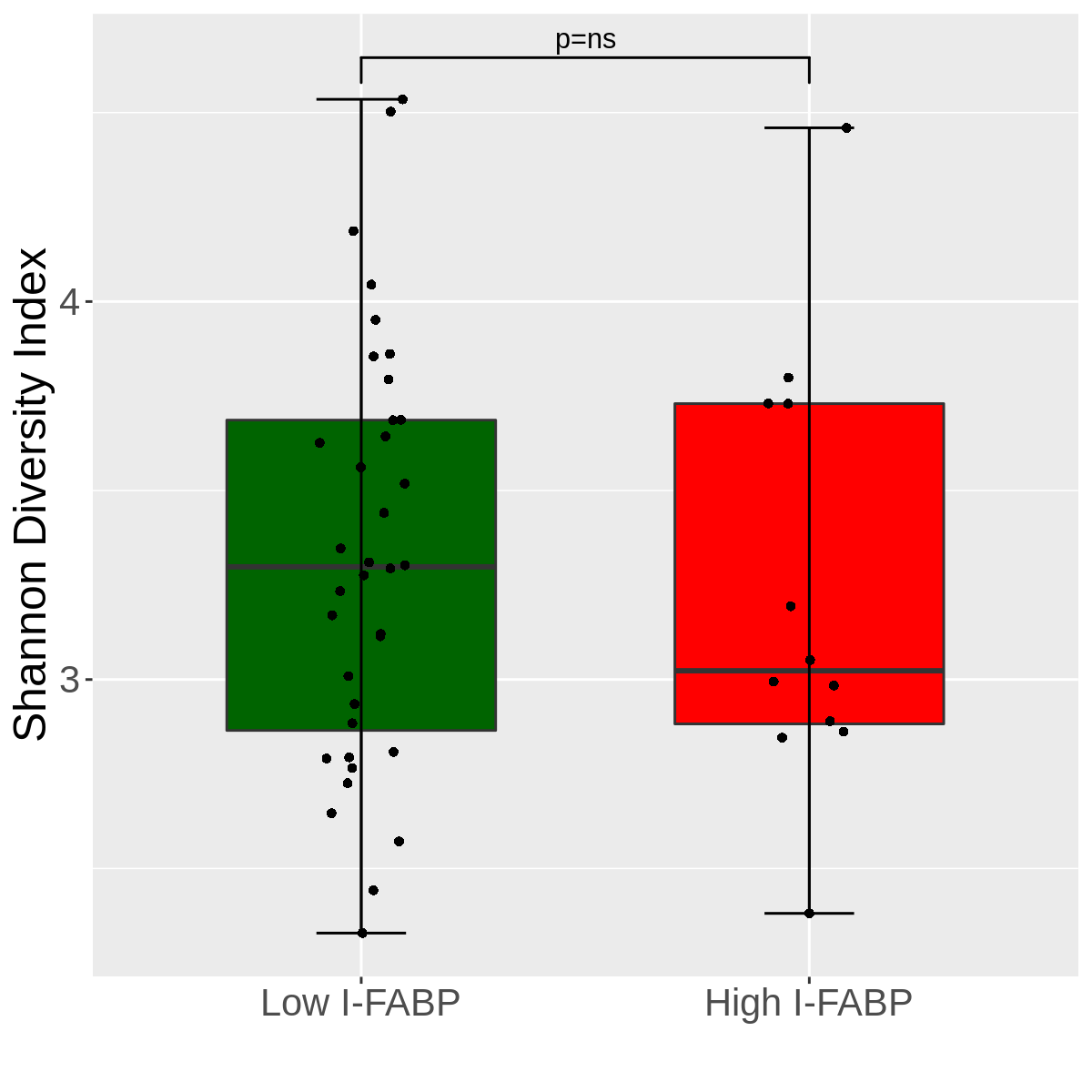

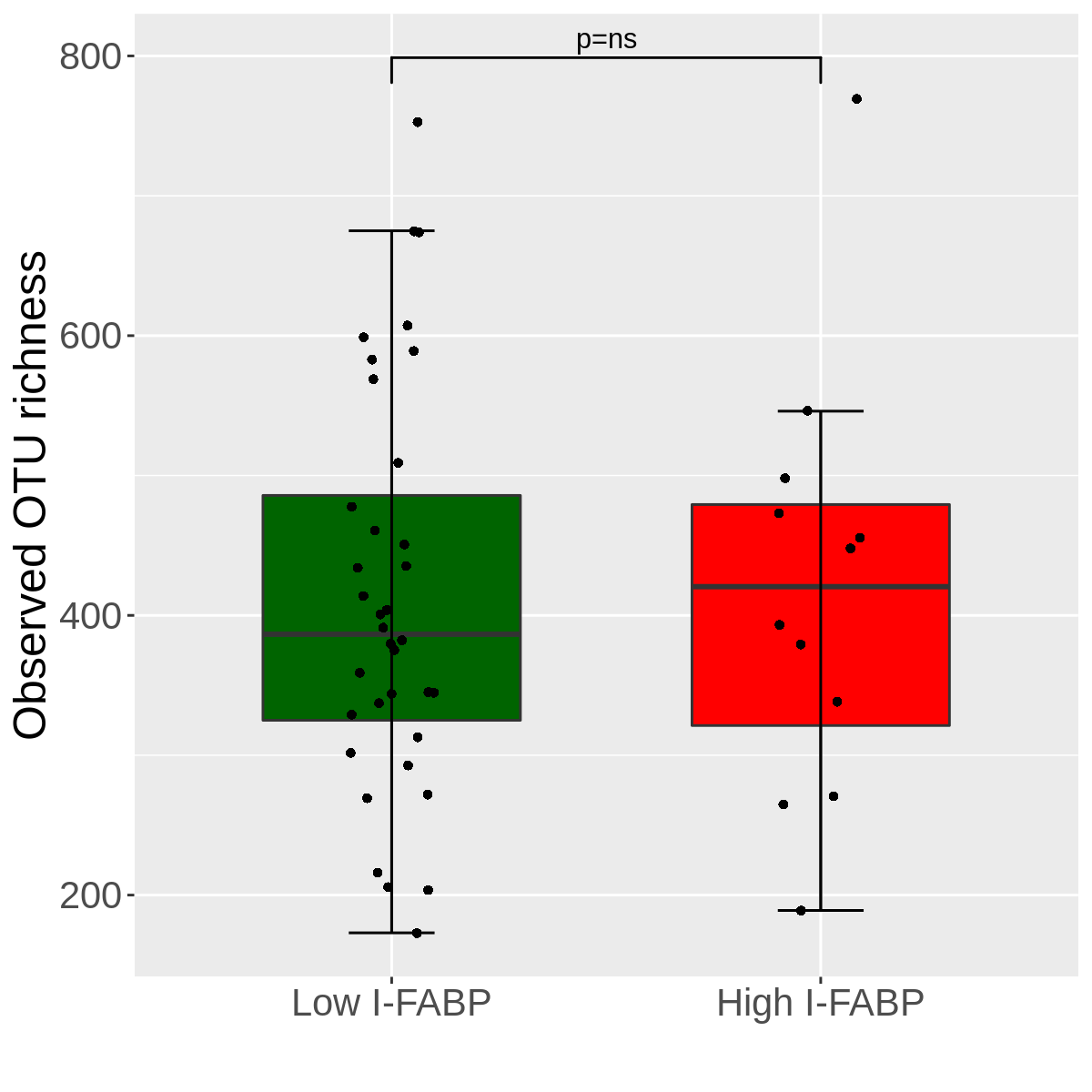


**Supplementary Figure S5. α diversity between high and low intestinal barrier markers.** α diversity differences in groups of (A) high vs. low D-LA (Wilcox *p*=ns). (B) high vs. low I-FABP (Wilcox *p*=ns).


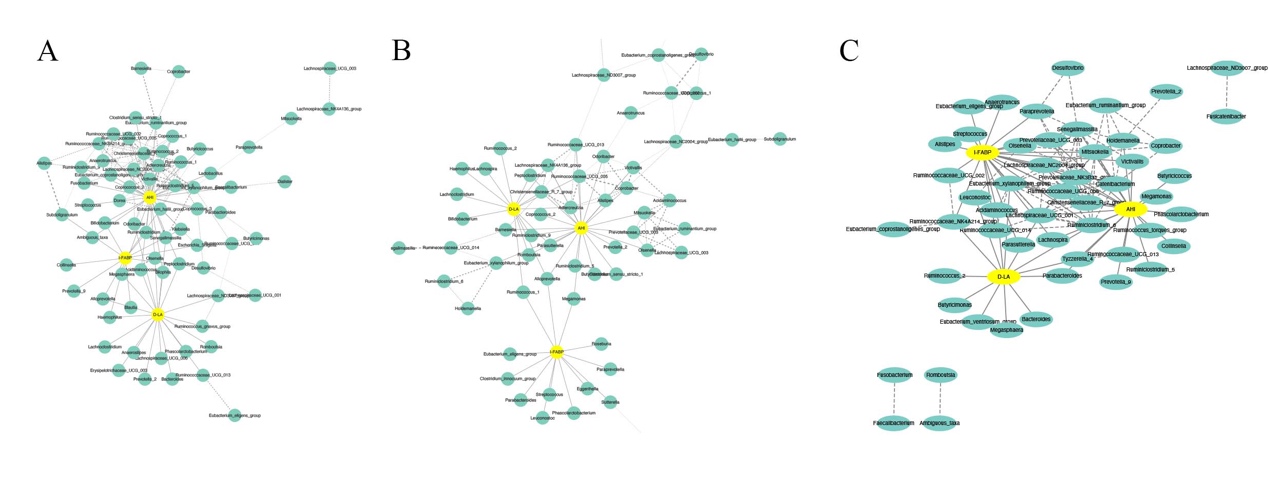


**Supplementary Figure S6.** The co-occurrence network at the genus level in (A) mild, (B) moderate, (C) heavy OSA.
