# Supplementary figures and images for: Obstructive sleep apnea is related to alterations in fecal microbiome and impaired intestinal barrier function
Source: Sci Rep. 2023 Jan 15;13:778. doi: 10.1038/s41598-023-27784-0 (PMC9841009; doi:10.1038/s41598-023-27784-0)

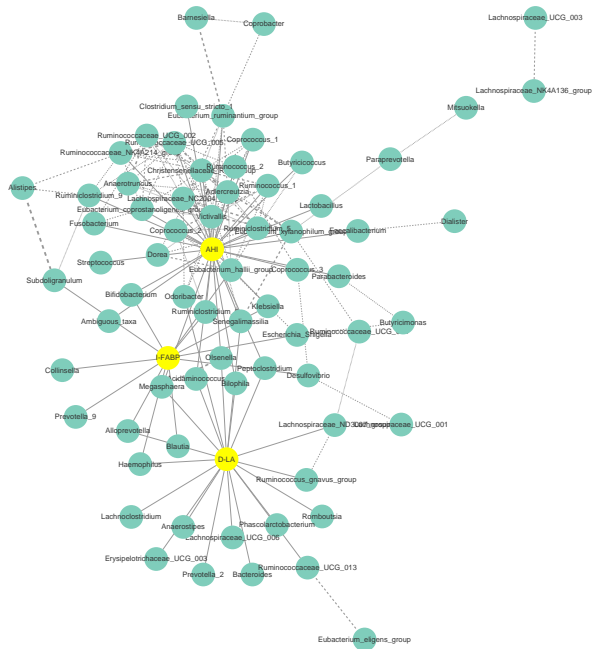

Supplement: Supplementary file 2 — Supplementary Information 2. [file 41598_2023_27784_MOESM2_ESM.pdf]

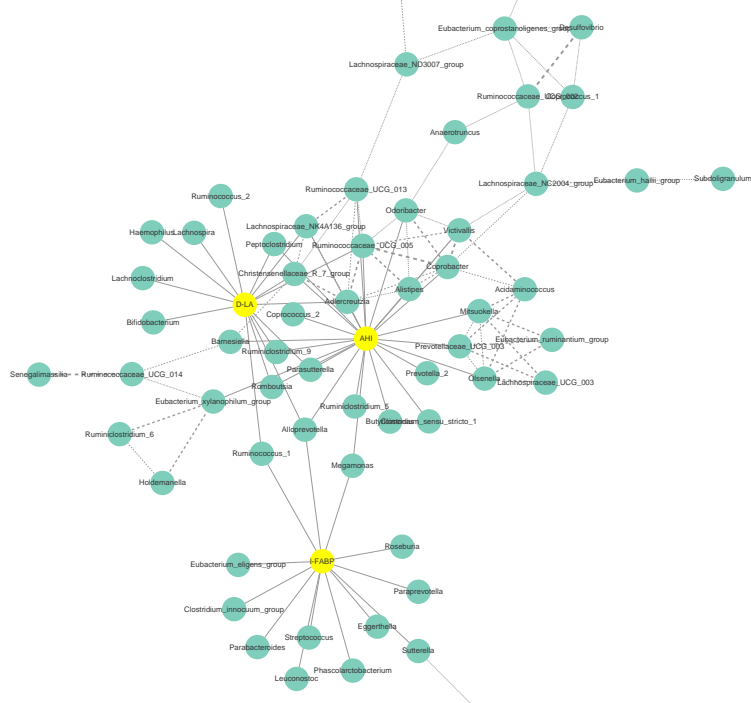

Supplement: Supplementary file 3 — Supplementary Information 3. [file 41598_2023_27784_MOESM3_ESM.pdf]

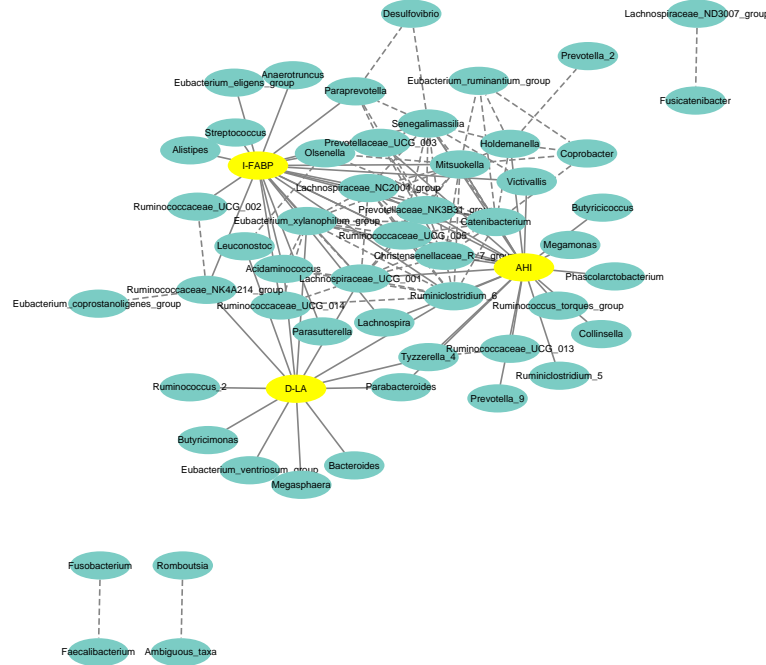

Supplement: Supplementary file 4 — Supplementary Information 4. [file 41598_2023_27784_MOESM4_ESM.pdf]
